# Reciprocal signaling-metabolic crosstalk between fibroblasts and tumor cells drives cetuximab tolerance in head and neck cancers

**DOI:** 10.1186/s13046-026-03765-9

**Published:** 2026-06-24

**Authors:** Engy Vigneron, Julie Vignau, Olivier Lowyck, Valentin Van den bossche, Jérôme Ambroise, Yann Kieffer, Fatima Mechta-Grigoriou, Isabella Rizzi, Maria Virginia Giolito, Manon Desgres, Hannah Zaryouh, An Wouters, Antonella Mendola, Hajar Dahou, Marine Leclerc, Romain Boidot, Aurélien Warnant, Yvan Larondelle, Olivier Feron, Jean-Pascal Machiels, Sandra Schmitz, Cyril Corbet

**Affiliations:** 1 Pole of Pharmacology and Therapeutics (FATH), Institut de Recherche Expérimentale et Clinique (IREC), UCLouvain, Avenue Hippocrate 57, B1.57.04, Brussels, B-1200 Belgium; 2 Pole of Molecular Imaging, Radiotherapy and Oncology (MIRO), Institut de Recherche Expérimentale et Clinique (IREC), UCLouvain, Avenue Hippocrate 57, Brussels, B-1200 Belgium; 3 Department of Medical Oncology, Institut Roi Albert II, Cliniques universitaires Saint-Luc, Avenue Hippocrate 10, Brussels, B-1200 Belgium; 4 Centre des Technologies Moléculaires Appliquées (CTMA), Institut de Recherche Expérimentale et Clinique (IREC), UCLouvain, Avenue Hippocrate 54, Brussels, B-1200 Belgium; 5 Stress and Cancer Laboratory, Equipe Labélisée Par La Ligue Nationale Contre Le Cancer, Inserm U1339 - UMR3666 CNRS, PSL Research University, Institute of Women’s Cancer, Institut Curie, 26, rue d’Ulm, Paris, F-75248 France; 6 Center for Oncological Research (CORE), Integrated Personalized & Precision Oncology Network (IPPON), University of Antwerp, Universiteitsplein 1, Antwerp, B- 2610 Belgium; 7 Unit of Molecular Biology, Department of Biology and Pathology of Tumors, Université Bourgogne Europe, Centre Georges-François Leclerc, Unicancer, ICMUB UMR CNRS 6302, Dijon, 21000 France; 8 Louvain Institute of Biomolecular Science and Technology (LIBST), UCLouvain, Croix du Sud 4-5/L7.07.03, Louvain-la-Neuve, B-1348 Belgium; 9 WEL Research Institute, avenue Pasteur 6, Wavre, B-1300 Belgium

**Keywords:** Head and neck cancer, Tumor microenvironment, Cancer-associated fibroblasts, Metabolism, Drug tolerance, Fatty acids, Cetuximab, Transforming growth factor beta

## Abstract

**Background:**

Resistance to EGFR-targeted therapies, including cetuximab, remains a major barrier to effective treatment of head and neck squamous cell carcinoma (HNSCC). Lipid metabolism reprogramming, TGFβ signaling, and cancer-associated fibroblast (CAF) activation have each been linked to cetuximab resistance, but how these processes mechanistically converge remains unclear.

**Methods:**

We integrated transcriptomic, metabolic, and biochemical assays in HNSCC cell lines, co-culture systems with matched patient-derived non tumoral fibroblasts and CAFs, patient-derived organoids (PDOs), and patient-derived xenografts (PDXs) to characterize tumor-stroma interactions during cetuximab treatment. Fluorescence-based fatty acid (FA) tracing and lipidomic analyses were used to assess FA flux. Functional relevance was tested through genetic and pharmacologic inhibition of TGF-β2 signaling and FA transfer. Clinical relevance was assessed by analyzing *TGFB2* expression and plasma TGF-β2 levels in HNSCC patient cohorts.

**Results:**

Cetuximab-treated HNSCC cells specifically upregulated and secreted TGF-β2, which induced two coordinated adaptive programs: (i) autocrine metabolic rewiring promoting FA uptake, oxidation, and storage in tumor cells, and (ii) paracrine activation of fibroblasts toward a myofibroblastic, lipid-secreting phenotype. These TGFβ2-activated CAFs supplied FA that sustained oxidative metabolism and preserved EGFR/MAPK signaling in tumor cells, establishing a reciprocal feedback loop that maintained a reversible drug-tolerant state. Disrupting either TGF-β2 signaling or FA transfer abrogated this circuit and restored cetuximab sensitivity in HNSCC cells, spheroids, and PDOs. In PDX models, TGF-β2 inhibition delayed tumor regrowth following cetuximab treatment. Elevated *TGFB2* expression and circulating TGF-β2 levels were associated with metabolic reprogramming and poorer clinical outcomes following cetuximab therapy.

**Conclusions:**

Our findings define a tumor-stroma metabolic cooperation axis in which TGFβ2-driven lipid exchange sustains adaptive cetuximab tolerance in HNSCC. Targeting TGFβ2-mediated stromal reprogramming or FA transfer represents a promising strategy to delay or overcome resistance to EGFR-targeted therapies.

**Graphical Abstract:**

**Figure:**
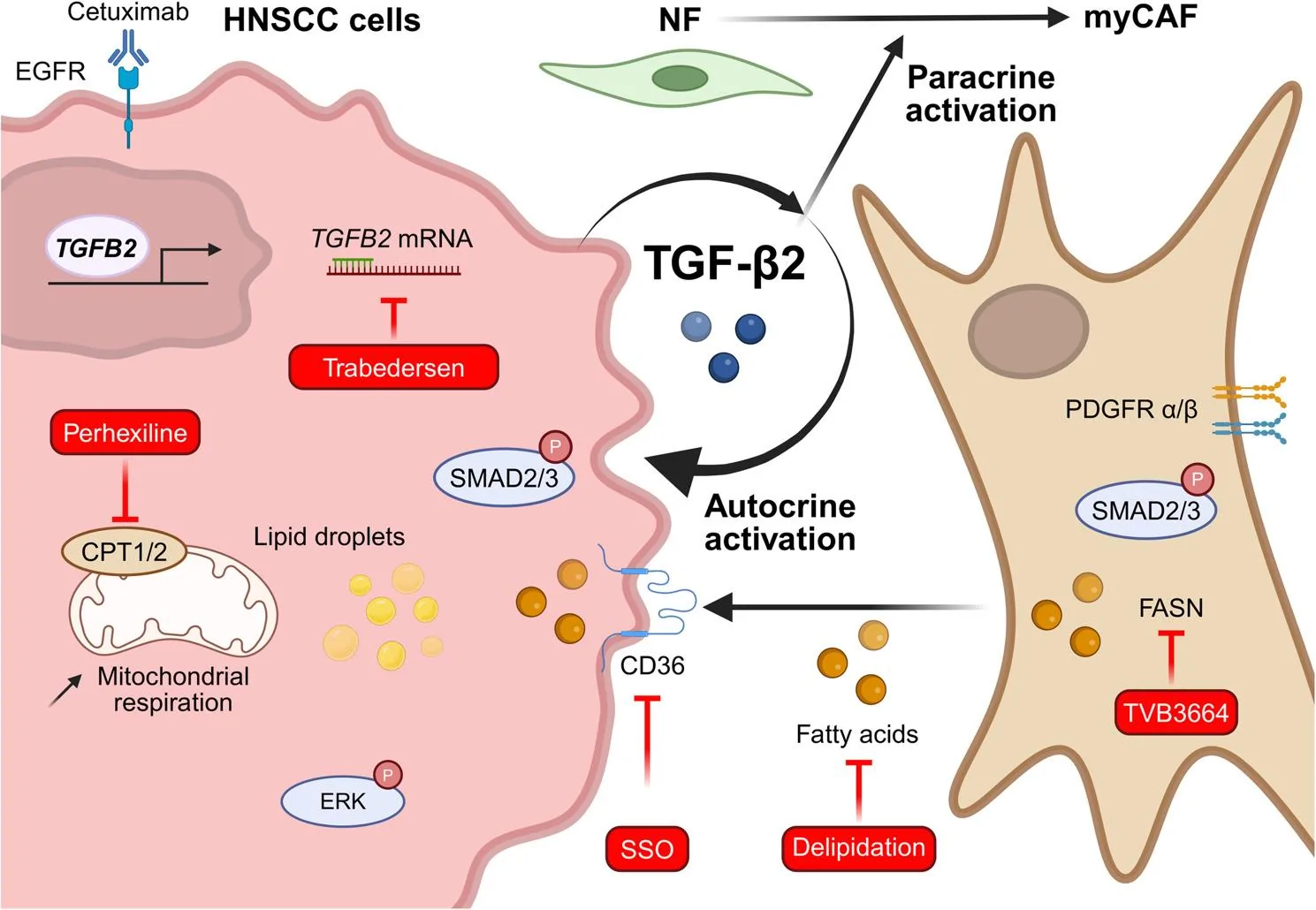

**Supplementary Information:**

The online version contains supplementary material available at https://doi.org/10.1186/s13046-026-03765-9.

## Background

Head and neck squamous cell carcinoma (HNSCC) ranks as the seventh most common cancer worldwide, with approximately 890,000 new cases and more than 450,000 deaths annually [1]. Despite advances in immunotherapy and anti-epidermal growth factor receptor (EGFR) targeted therapies, treatment options for locally advanced HNSCC have barely evolved over recent decades, and outcomes remain poor, particularly for HPV-negative disease and for patients with recurrent or metastatic tumors [2–4]. Cetuximab, the only FDA-approved EGFR-targeted monoclonal antibody, offers only modest and often transient clinical responses, with most patients eventually progressing due to the persistence of a drug-tolerant cell population [5, 6]. Across multiple cancer types, the depth of initial response to targeted therapy correlates with longer progression-free survival [7–9], highlighting the importance of residual disease as a driver of resistance. However, the molecular features enabling HNSCC cells to tolerate EGFR blockade before the acquisition of stable resistance remain incompletely understood.

Resistance to EGFR inhibition in HNSCC is multifactorial, encompassing PI3K/Akt pathway activation, compensatory signaling via alternative tyrosine kinase receptors, and metabolic reprogramming [10–12]. Most described mechanisms reflect acquired resistance, whereas early adaptive responses, particularly those involving tumor-microenvironment interactions, are less characterized. Among stromal components, cancer-associated fibroblasts (CAFs) constitute the predominant non-malignant population in the HNSCC tumor microenvironment (TME) [13], and their abundance correlates with advanced disease and poor prognosis [14, 15]. CAFs support tumor progression via extracellular matrix remodeling and paracrine signaling through cytokines, growth factors, exosomes, and miRNA [16–19]. However, whether EGFR-targeted therapy actively remodels the stromal compartment to support tumor cell persistence is still barely defined.

Here, we show that residual HNSCC cells adapt to EGFR blockade by specifically upregulating transforming growth factor beta 2 (TGF-β2), which promotes a metabolic shift towards fatty acid (FA) utilization in HNSCC cells and simultaneously reprograms fibroblasts towards a myofibroblastic, lipid-secreting phenotype. This reciprocal tumor-stroma interaction establishes a metabolic cooperation circuit that sustains cetuximab tolerance. Importantly, disruption of TGF-β2 signaling impairs stromal reprogramming, delays the emergence of cetuximab tolerance in preclinical models, and identifies TGFβ2-dependent tumor-stroma communication as a therapeutically actionable vulnerability in HNSCC.

## Methods

### Cell culture

HPV-negative human HNSCC cell lines SC263 and HN5 were provided by Prof. Sandra Nuyts (University Hospital Leuven, Leuven, Belgium) and Prof. Olivier De Wever (Ghent University Hospital, Ghent, Belgium). Human hypopharyngeal FaDu cells were purchased from ATCC (#HTB-43). Cells were stored according to the supplier’s instructions and used within 6 months after resuscitation of frozen aliquots. All cells were cultured in Dulbeccos’s modified Eagle’s medium (DMEM) containing 25 mM glucose and 4 mM GlutaMAX (#61965026, Thermo Fisher Scientific), supplemented with 10% heat-inactivated fetal bovine serum (FBS), 1% penicillin/streptomycin (Thermo Fisher Scientific) and maintained in exponential growth in 5% CO_2_/95% air in a humidified incubator at 37 °C. Cetuximab-resistant HNSCC cell populations (FaDu-R, HN5-R, and SC263-R) were generated by chronically exposing the parental cetuximab-sensitive cell lines to cetuximab until resistance ensued, as previously described [20–22], and then routinely cultured under continuous treatment with 15–30 µg/mL cetuximab (#L01XC06; Merck). All cell lines were routinely tested for mycoplasma contamination with the PCR-based MycoplasmaCheck service from Eurofins Genomics.

### Establishment of patient-derived fibroblast cultures

CAFs were isolated from treatment-naive, HPV-negative HNSCC patients undergoing biopsy or surgical resection at Cliniques universitaires Saint-Luc (Brussels, Belgium). Tissue samples were placed in RPMI 1640 medium (Gibco) supplemented with 0.4% fungizone (Bristol Myers Squibb), 2.5% penicillin/streptomycin (Sigma-Aldrich), and gentamycin (Braun Medical), and transported on ice within 45 min. After PBS washes, necrotic and non-viable tissue was excised, and portions of tumor tissue were snap-frozen (-80 °C) or fixed in 4% neutral-buffered formalin for histopathological analysis. The remaining tumor fragments were minced and dissociated enzymatically and mechanically using a GentleMacs OctoDissociator (Miltenyi Biotec) to obtain single-cell suspensions. The cells were cultured in 6-well plates and passaged twice prior to flow cytometry sorting based on negative expression of EpCAM, CD31, and CD45 markers and positive expression of CD90 on the surface to select the fibroblast population. CAFs were cultured in DMEM GlutaMAX containing 10% heat-inactivated FBS, 1% penicillin/streptomycin (Thermo Fisher Scientific), and 0.5 nM recombinant human basic fibroblast growth factor (#78003, STEMCELL Technologies). Non-tumoral fibroblasts (NFs) were cultured in human fibroblast expansion basal medium (M106500, Gibco) supplemented with 2% low serum growth supplement (#S00310, Thermo Fisher Scientific) and 1% penicillin/streptomycin. Both CAF and NF cultures were maintained at 37 °C in a humidified incubator (5% CO₂, 95% air). CAF were grown in tissue culture flasks pre-coated with collagen (#125 − 50, Sigma-Aldrich) according to the manufacturer’s instructions, whereas NF were cultured in uncoated flasks. All cultures were routinely tested for mycoplasma contamination using the MycoplasmaCheck PCR-based assay (Eurofins Genomics).

### Fibroblast-conditioned medium preparation

NFs or CAFs were seeded in collagen-coated T75 flask at a density of 2 × 10^6^ cells per flask. After 24 h, the culture medium was removed, and cells were washed twice with PBS before the addition of 11 mL serum-free DMEM supplemented with 1% penicillin/streptomycin. Following a 72-h incubation, conditioned medium (CM) was collected, centrifuged (1,500 rpm for 5 min at room temperature), and filtered through a 0.2-µm membrane. Cell numbers were determined manually using a Bürker hemocytometer for normalization purposes. CM was aliquoted (2 mL) into microtubes and stored at -80 °C.

For lipid depletion, CM was treated with fumed silica (#S5130, Sigma-Aldrich), as previously described [23]. Briefly, fumed silica powder was added to CM and incubated overnight at room temperature to allow precipitation. The supernatant was collected the following day, centrifuged, filtered through a 0.22-µm membrane, aliquoted, and stored at -80 °C.

### 2D cell culture treatment

CAF-CM or NF-CM was added in the culture medium to constitute 50% of the total volume. For the co-culture, HNSCC cancer cells were seeded in a companion 12-well plate and CAFs or NFs were added via a Transwell 0.4-µm pore polyester membrane insert, allowing for medium exchange while preventing cells from direct cell-cell interactions (#CLS3460, Corning). In some conditions, delipidated conditioned media were supplemented with 1% chemically defined lipid concentrate (#11905031, Thermo Fisher Scientific). Cell treatment was performed in routine culture medium with A922500 (#HY-10038, MedChemExpress), cetuximab (#L01XC06; Merck), GW6471 (#G5045; Sigma-Aldrich), perhexiline maleate (#HY-B1334A, MedChemExpress), recombinant human TGF-β2 (#HY-P7119, MedChemExpress), sulfo-N-succinimidyl oleate sodium (#HY-112847 A, MedChemExpress), Trabedersen (HY-142118, MedChemExpress), and TVB-3664 (#HY-120062, MedChemExpress) at different timings and concentrations, as indicated in the figure legends. Cell growth was assessed either by direct cell counting on a hemocytometer with Trypan Blue exclusion dye or by using the Presto Blue reagent (#A13262, Thermo Fisher Scientific) according to manufacturer’s instructions.

### Enzyme-linked immunosorbent assays

Active human EGF, TGF-α, and TGF-β2 protein levels were quantified in conditioned media from HNSCC cells and fibroblasts, organoid culture supernatants, and human plasma samples by using dedicated ELISA detection kits (#DEG00, R&D Systems for EGF; #EHTGFA, Thermo Fisher Scientific for TGF-α, and #DB250, R&D Systems for TGF-β2), following the manufacturer’s instructions. For TGF-β2 detection, samples were concentrated with Amicon Ultra-15 centrifugal filter units (#UFC903024, Merck) and then acid-activated, as previously described [24].

### 3D spheroid cultures

Spheroids were prepared with FaDu cells by seeding 1,000 cells/well and mixed spheroids were prepared with 500–750 FaDu cells and 750–1000 CAFs (ratio 1:1 to 1:2), in 96-well ultra-low attachment plates (Corning) in DMEM supplemented with 10% heat-inactivated FBS (or 10% delipidated FBS in some specific conditions), 25 mM D-glucose and 2 mM L-glutamine. Spheroids were treated with 1 µg/mL cetuximab, 3 days after seeding (day 0), in presence of NF-CM or CAF-CM (50%, v/v) in some conditions. Afterwards, the treatment was renewed every 2–3 days. Spheroid growth was monitored using live-cell phase contrast microscope (Axio Observer, Zeiss) and spheroid area was measured by using Fiji software [25]. Alternatively, cell viability (as an endpoint) was directly assessed by using the CellTiter-Glo^®^ 3D reagent (#G9681, Promega) according to manufacturer’s instructions.

### RNA extraction and RT-qPCR

Cell pellets were resuspended in Tri-Reagent (Molecular Research Center). RNA was recovered after separation in 1-bromo-3-chloropropane and precipitation with isopropanol, washed with 70% ethanol, resuspended in RNase-free water, and then quantified by spectrophotometry (Nanodrop 1000, Thermo Fisher Scientific). After reverse transcription on 1 µg of total RNA with the RevertAid Reverse-Transcriptase, oligo-dT and random hexamers (Thermo Fisher Scientific), quantitative PCR amplification was performed on a ViiA 7 real-time PCR system (Applied Biosystems) using SYBR^®^ select master mix for cfx (Thermo Fisher Scientific) and gene-specific primer sequences (Supplementary Table 1). Data were analyzed according to ddCt method using human *GTF2B* as reference gene.

### RNA sequencing

Total RNA was extracted from cells (HNSCC cells and fibroblasts) using the Monarch Total RNA Miniprep Kit (New England Biolabs). Ribosomal RNA (rRNA) was depleted using the RiboCop rRNA Depletion Kit (Lexogen). The rRNA-depleted RNA was then used for library preparation with the NEBNext Ultra II Directional RNA Library Prep Kit for Illumina (New England Biolabs), following the manufacturer’s instructions. RNA sequencing was performed on an AVITI platform (Element Biosciences). The RNA-seq libraries were sequenced using 76 bp paired-end reads, and samples were multiplexed to achieve 20 million reads per sample.

For PDX samples, RNA extraction and sequencing was carried out by IntegraGen. RNeasy^®^ Plus Mini Kit (Qiagen) was used for purification of total RNA from tissues, using gDNA Eliminator columns. Each RNA sample was loaded on Fragment Analyzer (Agilent) for a precise quantification and quality control. Libraries were prepared with NEBNext^®^ Ultra™ II Directional RNA Library Prep Kit for Illumina protocol according supplier recommendations. Briefly the key stages of this protocol are successively, the purification of PolyA containing mRNA molecules using poly-T oligo attached magnetic beads from 100ng total RNA (with the Magnetic mRNA Isolation Kit from NEB), a fragmentation using divalent cations under elevated temperature to obtain approximately 300 bp pieces, double strand cDNA synthesis and finally Illumina adapters ligation and cDNA library amplification by PCR for sequencing. The sequencing was performed on an Illumina NovaSeq X Plus sequencer as paired end 2 × 100 bp reads.

### RNA-seq and tiling array data analysis

The RNA sequencing data were analyzed using the Automated Reproducible MOdular workflow for preprocessing and differential analysis of RNA-seq data (ARMOR v1.5.4) [26]. We performed quality control on reads with FastQC and quantified them using Salmon [27] against a transcriptome index built from all Homo sapiens Ensembl cDNA sequences (GRCh38). Quality control summaries were generated using MultiQC. Transcript abundances from Salmon were then imported into R (v4.1.1) via the tximeta package [28] and analyzed for differential gene expression (DGE) using edgeR [29]. For the PDX samples, sequencing reads were simultaneously mapped to both the human (GRCh38) and mouse (Mus musculus GRCm39) genomes using STAR v2.7.10. A count matrix of human-specific reads was generated using XenofilteR [30], imported into R, and subsequently analyzed with edgeR (v.4.6.3) to identify DGE. The tiling array expression data (GSE109756) were also subjected to DGE analysis using the limma package (v.3.64.3) [31] to compare post-cetuximab versus baseline samples. Enrichment analysis was performed with the fgsea package (v.1.35.6) to produce an enrichment plot for the hallmark oxidative phosphorylation (OXPHOS) pathway.

### Deconvolution of bulk RNA-seq data

Bulk RNA-seq from five matched CAF/NF pairs (3 replicates each) were deconvolved using BayesPrism algorithm version v2.2.2. As prior information, we used a raw count matrix comprising 15,667 CAF-S1 single cells (annotated as Detox-iCAF, IL-iCAF, IFNg-iCAF, ECM-myCAF, TGFb-myCAF, Wound-myCAF, and IFNab-myCAF) and normal fibroblasts (annotated as Fibroblasts and Universal fibroblasts) [32]. Gene markers used to define each fibroblast subtype are listed in Supplementary Table 2. To improve specificity, mitochondrial and ribosomal protein-coding genes were excluded, as their high expression levels are not informative for distinguishing cell types. Following the recommendations of BayesPrism’s authors, MALAT1 and genes located on chromosomes X and Y were also removed. To mitigate batch effects and accelerate computation, the deconvolution was restricted to protein-coding genes only. Gibbs sampling and optimization were performed using default BayesPrism parameters. Final estimates of cell type fractions were obtained from the updated theta matrix and subsequently used for downstream analyses.

### Fatty acid quantification

Conditioned media from NF and CAF were collected for total lipid extraction using a methanol: chloroform: water (2:2:1.8; v: v:v) protocol. Internal standards were added to each sample prior to extraction to assess lipid recovery and fractionation efficiency. The internal standard mixture included nonadecanoic acid (free FA), 1,2-dipentadecanoyl-sn-glycero-3-phosphatidylcholine (phospholipids), and triheptadecanoin (neutral lipids) (Larodan). Following extraction, samples were dried under nitrogen at 30 °C and resuspended in chloroform. Lipid classes were separated by solid phase extraction columns using Bond Elut-NH_2_ columns (Agilent Technologies). Neutral lipids, free FA, and phospholipids were sequentially eluted with chloroform:2-propanol (2:1; v: v), diethyl ether: acetic acid (98:2; v: v), and methanol, respectively. Eluted fractions were dried under a nitrogen flux at 30 °C and subjected to a two-step methylation process: alkaline methylation with 0.5 mL of 0.1 M KOH in methanol at 70 °C for 1 h with vigorous shaking every 15 min, followed by acidic methylation through the addition of 0.2 mL of 1.2 M HCl in methanol and incubation at 70 °C for 15 min. FA methyl esters (FAMEs) were then extracted using hexane. Methyl-undecanoate (Larodan) was added as an injection standard before analysis. FAMEs were separated by gas chromatography column using a Trace 1310 GC system equipped with a TriPlus AS autosampler and an RT-2560 capillary column (100 m x 0.25 mm internal diameter, 0.2 μm film thickness; Restek). The carrier gas was hydrogen (200 kPa constant flow). The temperature program was as follows: 80 °C ramped to 175 °C at 25 °C/min (held for 25 min), then to 200 °C at 10 °C/min (held for 20 min), followed by 220 °C at 10 °C/min (held for 5 min), and finally to 235 °C at 10 °C/min (held for 15 min). The oven was cooled back to 80 °C at 20 °C/min. Detection was performed using a flame ionizing detector maintained at 255 °C with the following gas flows: air (350 mL/min), hydrogen (35 mL/min) and nitrogen (40 mL/min). FAMEs were identified and quantified by comparing retention times and peak areas to those of an external standard mix comprising 43 pure methyl esters (Larodan; Nu-Check Prep). Data were acquired and processed using ChromQuest 5.0 software (Thermo Fisher Scientific). FA concentrations were normalized to internal and injection standards and expressed as nmol FA per 10^6^ cells.

### Western blot analysis

Subconfluent cancer cells were washed twice with ice-cold PBS and lysed in a RIPA buffer supplemented with a protease inhibitor cocktail (Sigma-Aldrich) and a phosphatase inhibitor cocktail (Roche). Cell lysates were then cleared by centrifugation (6,000 x g, 10 min, 4 °C) and stored at -80 °C until analysis. After determination of protein concentration using a bicinchoninic acid-based assay (Thermo Fisher Scientific), samples were denaturated (5 min, 95 °C) with Laemmli sample buffer containing 100 mM dithiothreitol. Samples (20–40 µg per well) were then separated by SDS-PAGE (8 to 15% acrylamide/bis-acrylamide gels) and transferred to PVDF membranes. Membranes were blocked with 5% bovine serum albumin (BSA) in TBS-0.1% Tween 20 (TTBS) and subsequently immunoblotted overnight at 4 °C with specific primary antibodies against β-actin (clone AC-15, #A5441, Sigma-Aldrich; 1:10,000), phosphorylated Smad2 (Ser-465/467, clone 138D4, #3108, Cell Signaling Technology; 1:1,000) phosphorylated Smad3 (Ser-423/425; clone C25A9, #9520 Cell Signaling Technology; 1: 10419, 1,000), total Smad2/3 (clone D7G7, #8685, Cell Signaling Technology; 1:1,000), Hsp90 (clone 68, #6 BD Biosciences; 1:7,500), PDGFRα (clone D1E1E, #3174, Cell Signaling Technology; 1:1,000), PDGFRβ (clone 28E1, #3169, Cell Signaling Technology; 1:1,000), and FAP (clone E1V9V, #66562, Cell Signaling Technology; 1:1,000), phosphorylated p44/42 MAPK (Erk1/2) (Thr202/Tyr204, clone D13.14.4E, #4370, Cell Signaling Technology; 1:1,000), total p44/42 MAPK (Erk1/2) (clone 137F5, #4695, Cell Signaling Technology; 1:1,000), phosphorylated Akt (Ser473, clone D9E, #4060, Cell Signaling Technology; 1:1,000), total Akt (#9272, Cell Signaling Technology; 1:1,000). After several washes with TTBS, membranes were then incubated (1 h, room temperature) with horseradish peroxidase (HRP)-conjugated secondary antibodies (#115-035-003 and #111-035-003, Jackson Immunoresearch; 1:10,000) and chemoluminescent signals were revealed by using ECL Western Blotting Detection Kit (GE Healthcare) on X-ray films in a dark chamber. Films were scanned with an Epson V600 scanner and images were processed with ImageJ.

### Seahorse analysis

All assays were carried out using a seeding density of 30,000 cells/well in Seahorse Bioanalyzer XFe96 culture plates in non-buffered DMEM, adjusted at pH 7.4 and supplemented with specified metabolic substrates. To evaluate FA oxidation capacity, cells were incubated in DMEM containing 50 µM palmitate-BSA (Agilent). Oxygen consumption rate (OCR) was assessed for 3 cycles (3 min mixing/4 min measuring). Data were normalized by the protein content in each well and expressed in pmoles/min/µg protein (OCR). OCR were measured with Seahorse XFe96 Analyzer (Agilent) and data analysis was performed using Seahorse Analytics software (version 1.0.0-768).

### Flow cytometry

For palmitate uptake analysis, HNSCC cells were washed with PBS and incubated with 0.2 µM BODIPY™ FL C16 (#D3821; Thermo Fisher Scientific) for 15 min at room temperature. Cells were washed three times with ice-cold PBS, fixed with 4% paraformaldehyde (PFA, #1004969011 Sigma Aldrich) for 30 min at room temperature, and analyzed on a BD FACSCanto™ II flow cytometer (BD Biosciences) using the FL1-FITC channel. For fibroblast population phenotyping, cells were washed twice with PBS and incubated for 30 min at 4 °C with the following antibodies: PE/Cyanine7-conjugated anti-human CD326 (EpCAM; clone 9C4, #324221, BioLegend), APC anti-human CD31 (clone WM59, #303115, BioLegend), PE anti-human CD90 (Thy1; clone 5E10, #328109, BioLegend), and FITC anti-human CD45 (clone HI30, #304005, BioLegend) (1 µL per 10^6^ cells for each antibody), together with 0.5 µM DAPI (#D9542; Sigma-Aldrich) in FACS tubes (#CLS352008; Sigma-Aldrich). Cells were washed twice with PBS, resuspended in 200 µL PBS, and analyzed on a BD FACSCanto™ II flow cytometer using the FL1-FITC, FL2-APC, FL3-CD90, FL4-PE-Cy7, and FL5-Pacific Blue channels. For all assays, at least 10,000 events were recorded per sample. Data were analyzed using FACSDiva v7 and FlowJo v10.7.1 softwares (BD Biosciences).

### Oil red-O staining

HNSCC cancer cells were seeded in a 12-well microscopy slide with removable chamber (#81201, Ibidi) and allowed to reach 50%-75% confluence under treatment. A stock solution of 150 mg Oil Red-O (ORO, #00625; Sigma Aldrich) in 50 mL isopropanol prepared in advance was freshly diluted in demineralized water (2/5 v/v). Cells were rinsed twice with PBS and fixed with 4% PFA solution for 20 min at room temperature. After several washes with PBS and pre-incubation with 60% (v: v) isopropanol during 5 min, the working solution of ORO was added for 30 min. Cells were washed 3 times with tap water and nuclear staining was performed with hematoxylin during 5 min. After several washes with tap water, slides were mounted with mounting medium (#S3025; Agilent Dako). Brightfield pictures were taken with a Zeiss Axio Imager microscope and quantification of lipid droplets was done using Fiji [25]. Treatment with 50 µM BSA-conjugated oleic acid (Larodan) was used as a positive control for lipid droplet accumulation.

### In vivo mouse experiments

All mice were housed in the animal facility of the Institut de Recherche Expérimentale et Clinique (IREC, UCLouvain) under controlled environmental conditions, including a 12 h light/12 h dark cycle, temperature of 21–24 °C, and relative humidity of 40–60%. Mice had ad libitum access to water and a complete maintenance diet (#U8220G10R, SAFE). HNC004 and UCLHN4 PDX models were established from treatment-naive HPV-negative HNSCC patients, as previously described [12, 33, 34]. For all in vivo experiments, 7-week-old female Rj: NMRI-*Foxn1*^*nu/n*^*u* mice were purchased from Janvier Labs. For the in vivo co-injection experiments, 24 mice were subcutaneously injected in the cervical region with a mixture of HNSCC cells and fibroblasts. FaDu cells were washed twice with PBS and resuspended in 0.9% NaCl together with either CAFs or NFs (HT15B model). A total of 3 × 10^6^ cells (FaDu: fibroblast, ratio 1:2) suspended in 100 µL of 0.9% NaCl were injected per mouse. Once tumors reached a mean diameter of 5 mm, mice received weekly intraperitoneal injections of cetuximab (30 mg/kg in 0.9% NaCl). Control groups were treated in parallel with adequate vehicle. Mice were weighed and tumors were measured 3 times a week. For PDX experiments, 24 mice were implanted subcutaneously with UCLHN4 PDX tumor fragments derived from frozen tumors and resuspended in Cultrex BME Type 2 (#3532-010-02, Bio-Techne). When tumors reached a mean diameter of 5 mm, mice were randomized to receive intraperitoneal injections twice weekly of cetuximab (30 mg/kg in 0.9% NaCl), Trabedersen (50 mg/kg for the first dose followed by 16 mg/kg for subsequent doses in 0.9% NaCl) or the combination treatment consisting of Trabedersen resuspended in the cetuximab solution. Control mice receive the appropriate vehicle. Tumor dimensions and body weight were monitored throughout the study. Tumor volumes did not exceed the limits approved by the local ethical committee. Mice were euthanized when tumors reached the predefined ethical endpoint (tumor volume ≥ 1500 mm³ or evidence of visible necrosis), and tumors were subsequently harvested for downstream analyses.

### Tissue processing and organoid culture

HNSCC organoids were established from the UCLHN4 PDX model. Fresh tumor tissue was collected in Advanced DMEM/F12 (Gibco) supplemented with 10 mM HEPES (#15630056, Thermo Fisher Scientific), L-glutamine (#25030024, Thermo Fisher Scientific), penicillin-streptomycin (#15140122, Thermo Fisher Scientific), 100 µg/mL primocin (#ant-pm-1, InvivoGen), B-27 supplement without vitamin A, 1× (#12587010, Thermo Fisher Scientific), and 10 µM Y-27,632 (#72302, STEMCELL Technologies). Samples were transported on ice and processed within 45 min of collection. Tumor specimens were washed twice with PBS, mechanically minced into small fragments, and enzymatically dissociated using the Tumor Dissociation Kit, human (#130-095-929, Miltenyi Biotec) on a gentleMACS Octo Dissociator (program 37C_h_TDK_2, 35 min; Miltenyi Biotec). Enzymatic digestion was stopped by adding Advanced DMEM/F12 supplemented with L-glutamine and penicillin-streptomycin. Cells were subsequently washed twice with cold PBS to obtain a single-cell suspension containing minimal residual tissue fragments.

Organoids were cultured in Advanced DMEM/F12 supplemented with 10 mM HEPES, L-glutamine, penicillin-streptomycin, 100 µg/mL primocin, B-27 without vitamin A (1X), 10 µM Y-27,632, 100 mM nicotinamide (#N0636, Sigma-Aldrich), 1.25 mM N-acetylcysteine (#A9165, Sigma-Aldrich), 500 nM A-83-01 (#72022, STEMCELL Technologies), 1 µM forskolin (#F3917, Sigma-Aldrich), 300 nM CHIR99021 (#SML1046, Sigma-Aldrich), 250 ng/mL human recombinant R-spondin-1 (#120 − 38, Thermo Fisher Scientific), 5 ng/mL basic FGF (#78003.1, STEMCELL Technologies), 10 ng/mL human recombinant FGF-10 (#78037, STEMCELL Technologies), 50 ng/mL human recombinant EGF (#78006.1, STEMCELL Technologies), 1 µM prostaglandin E2 (#72192, STEMCELL Technologies), and 100 ng/mL human recombinant noggin (#120–10 C, Thermo Fisher Scientific). Cells were embedded in 50-µL domes of Cultrex BME Type 2 (#3532-010-02, Bio-Techne) in 24-well plates (#3524, Corning Costar). For functional assays, organoids were cultured for 2 weeks in 5-µL BME domes in 96-well plates, with medium replaced every 2–3 days. Organoids were treated with vehicle, cetuximab (2 µg/mL), or Trabedersen (10 µM), and in the presence or absence of CM from CAFs or NF. Fibroblasts were either untreated or preconditioned with CM collected from Trabedersen-treated FaDu cells. Organoid viability was assessed at the experimental endpoint using the CellTiter-Glo^®^ 3D assay kit (#G9681, Promega) according to manufacturer’s instructions. Cytotoxicity was evaluated using the CellTox™ Green (#G8741, Promega) by adding 21 µL of reagent to 200 µL of culture medium and incubating for 24 h prior to fluorescence measurement (Ex 500 nm/ Em 530 nm). Fluorescence values were normalized to DNA content using the QuantiFluor^®^ dsDNA system (#E2670, Promega) according to the manufacturer’s instructions. For Western blot analyses, organoids were cultured for 2 weeks in 50-µL BME domes in 24-well plates using lacking A-83-01, refreshed every 2–3 days, and treated with or without cetuximab (2 µg/mL). Organoids from multiple wells were pooled, washed five times with ice-cold PBS, and lysed in RIPA buffer supplemented with protease (Sigma-Aldrich) and phosphatase (Roche) inhibitor cocktails. Samples underwent three freeze-thaw cycles at -20 °C, followed by sonication in a 40-Hz ultrasonic bath (ATM40-3LCD, Led Techno) for 5 min. Mechanical homogenization was then performed using 1.4 mm zirconium oxide beads (#P000927-LYSK0-A, Bertin-Technologies) in a Precellys^®^ Evolution Touch homogeneizer (Eqs. 02520-300-RD000, Bertin-Technologies) for six cycles of 30 s at 7,800 rpm.

### Statistics and reproducibility

Statistical analyses were performed using GraphPad Prism 10. Comparisons were conducted using Student’s *t*-test, one-way ANOVA with Dunnett’s multiple comparison test, two-way ANOVA with Tukey’s or Sidák’s multiple comparison test, as appropriate. Exact *P*-values are reported when *P* < 0.05 or *P* < 0.01, while ***P < 0.001 indicates a threshold value. Sample sizes were based on standard practices in the field and were not predetermined statistically. No data were excluded. Mice were randomly assigned to treatment groups; other experiments were not randomized. Investigators were not blinded during data collection or analysis.

## Results

### Residual HNSCC cells upregulate TGF-β2 in response to EGFR blockade

**Fig. 1:**
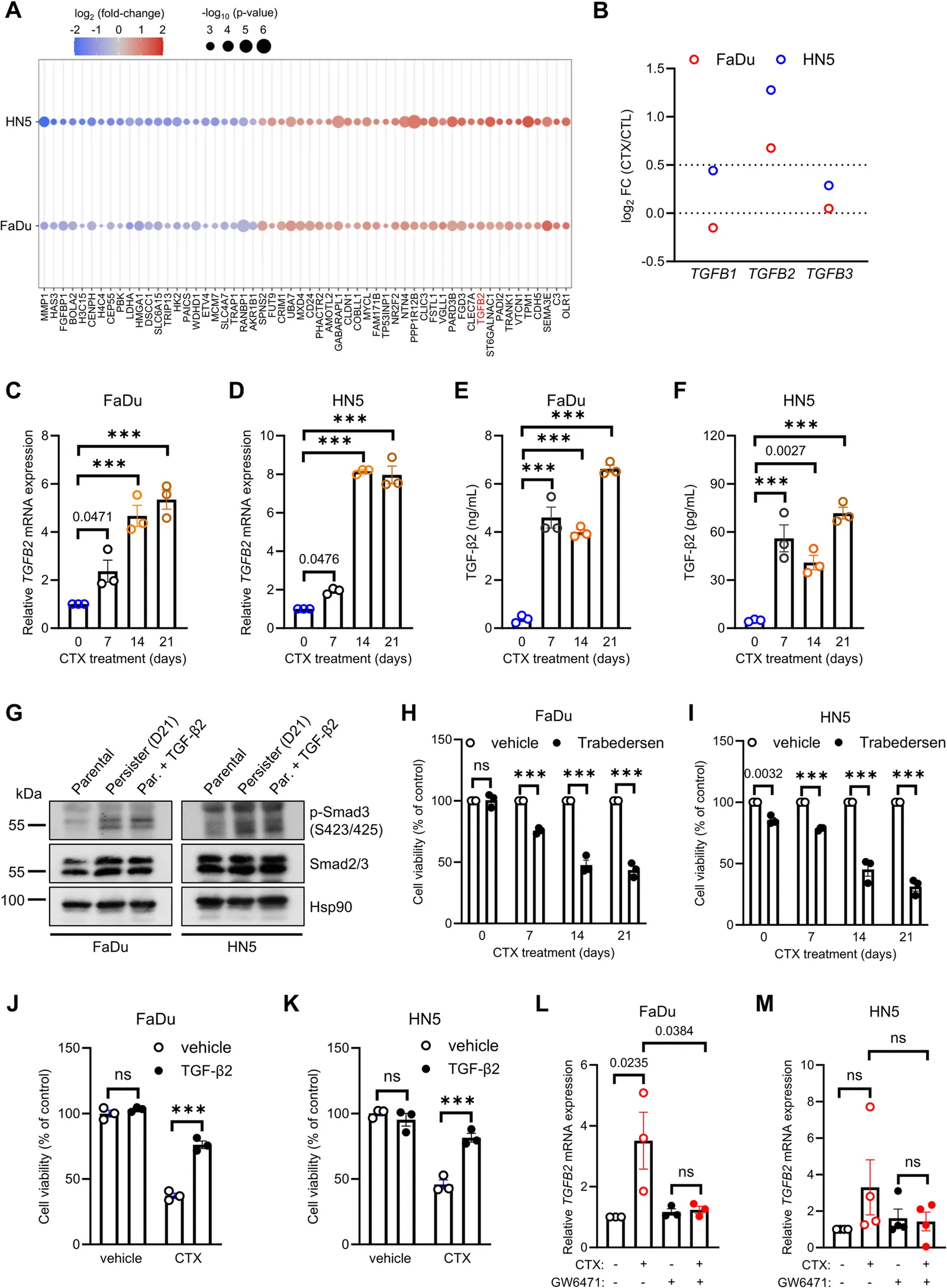
Residual HNSCC cells upregulate TGF-β2 under anti-EGFR therapy. **A** Dot plots depicting the number of genes commonly down- or up-regulated in RNA-seq analysis (GSE306480) of FaDu and HN5 cells after treatment with 1 µg/mL cetuximab for 72 h (*N* = 3). **B** Relative mRNA expression (log_2_ fold-change) of *TGFB1*, *TGFB2* and *TGFB3* in cetuximab-treated FaDu and HN5 cells (*N* = 3). **C-D** mRNA expression levels of TGF-β2 in FaDu **C** and HN5 **D** cells treated with 1 µg/mL cetuximab for 7, 14, and 21 days (*N* = 3; *n* = 2–3). **E-F** Active TGF-β2 protein levels in conditioned media from FaDu **E** and HN5 **F** cells treated with 1 µg/mL cetuximab for 7, 14, and 21 days (*N* = 3; *n* = 1–2). **G** Representative immunoblot for phosphorylated and total forms of Smad3 in FaDu cells treated with 1 µg/mL cetuximab for 21 days or with 10 ng/mL TGF-β2 for 6 h (*N* = 3). **H-I** Viability of FaDu **H** and HN5 **I** cells after treatment with 1 µg/mL cetuximab for 7, 14, or 21 days, and then incubation with 10 µM Trabedersen for 7 days (*N* = 3; *n* = 6). **J-K** Viability of FaDu **J** and HN5 **K** cells after treatment with 10 ng/mL TGF-β2 for 48 h and then incubation with 1 µg/mL cetuximab for 72 h (*N* = 3; *n* = 6). **L-M** mRNA expression levels of TGF-β2 in FaDu **L** and HN5 **M** cells treated with 1 µg/mL cetuximab and 10 µM GW6471 for 72 h (*N* = 3–4; *n* = 3). Data are plotted as the means ± SEM **C-F** and **H-M**. *N* indicates the number of independent biological experiments and *n* indicates the number of technical replicates (when > 1). Significance was determined by one-way ANOVA with Dunnett’s multiple comparison test **C-F** and **L-M**, or two-way ANOVA with Tukey’s multiple comparison test **H-K**. *P*-values as indicated or ***P < 0.001; ns, not significant

To uncover early transcriptional adaptations to EGFR inhibition, we performed bulk RNA-sequencing on FaDu and HN5 cells treated with cetuximab for 72 h (Supplementary Figures S1A-C). Differential gene expression analysis revealed 33 genes consistently upregulated across both cell lines, among which *TGFB2* emerged as a top candidate due to its established role in tumor-stroma crosstalk (Fig. 1A). Upregulation was isoform-specific, as *TGFB1* and *TGFB3* transcript levels remained unchanged (Fig. 1B). Sustained TGF-β2 induction at the mRNA and protein levels was confirmed up to 21 days after treatment initiation (Figs. 1C-F and Supplementary Figure S1D), coinciding with increased Smad2/3 phosphorylation, indicative of TGF-β pathway activation (Fig. 1G and Supplementary Figure S1E). Despite prolonged cetuximab exposure, cell numbers remained stable (Supplementary Figure S1F), consistent with a drug-tolerant persister state that remained reversible upon drug withdrawal and rechallenge (Supplementary Figure S1G).

To determine whether TGF-β2 contributes functionally to cetuximab tolerance, we suppressed *TGFB2* expression using Trabedersen, a specific antisense oligonucleotide. Trabedersen significantly reduced the viability of cetuximab-treated persister HNSCC cells but had no effect on untreated parental cells (Figs. 1H-I and Supplementary Figure S1H). Conversely, treatment with recombinant TGF-β2 induced a cetuximab-tolerant cell phenotype (Figs. 1J-K and Supplementary Figure S1I). Elevated TGF-β2 levels and phospho-Smad2/3 were also observed in HNSCC cell lines with acquired resistance to cetuximab (upon long-term drug exposure) (Supplementary Figures S1J-L). Together, these findings support the existence of a TGFβ2-dependent autocrine loop that actively sustains cetuximab tolerance in HNSCC cells.

To investigate the molecular mechanism underlying cetuximab-induced *TGFB2* expression, we examined the potential involvement of PPARα, a lipid-sensing nuclear receptor previously implicated in FA metabolic adaptation during cetuximab resistance^12^. Pharmacological inhibition of PPARα with the selective antagonist GW6471 significantly decreased *TGFB2* mRNA expression in HNSCC cells (Figs. 1L-M and Supplementary Figure S1M), demonstrating that PPARα activity is required, at least in part, for TGF-β2 induction upon EGFR blockade. These findings are consistent with previous mechanistic studies reporting a positive association between PPARα expression and *TGFB2* levels, and induction of TGF-β2 following fibrate treatment [35].

### EGFR blockade induces a TGFβ2-dependent shift toward FA metabolism

**Fig. 2:**
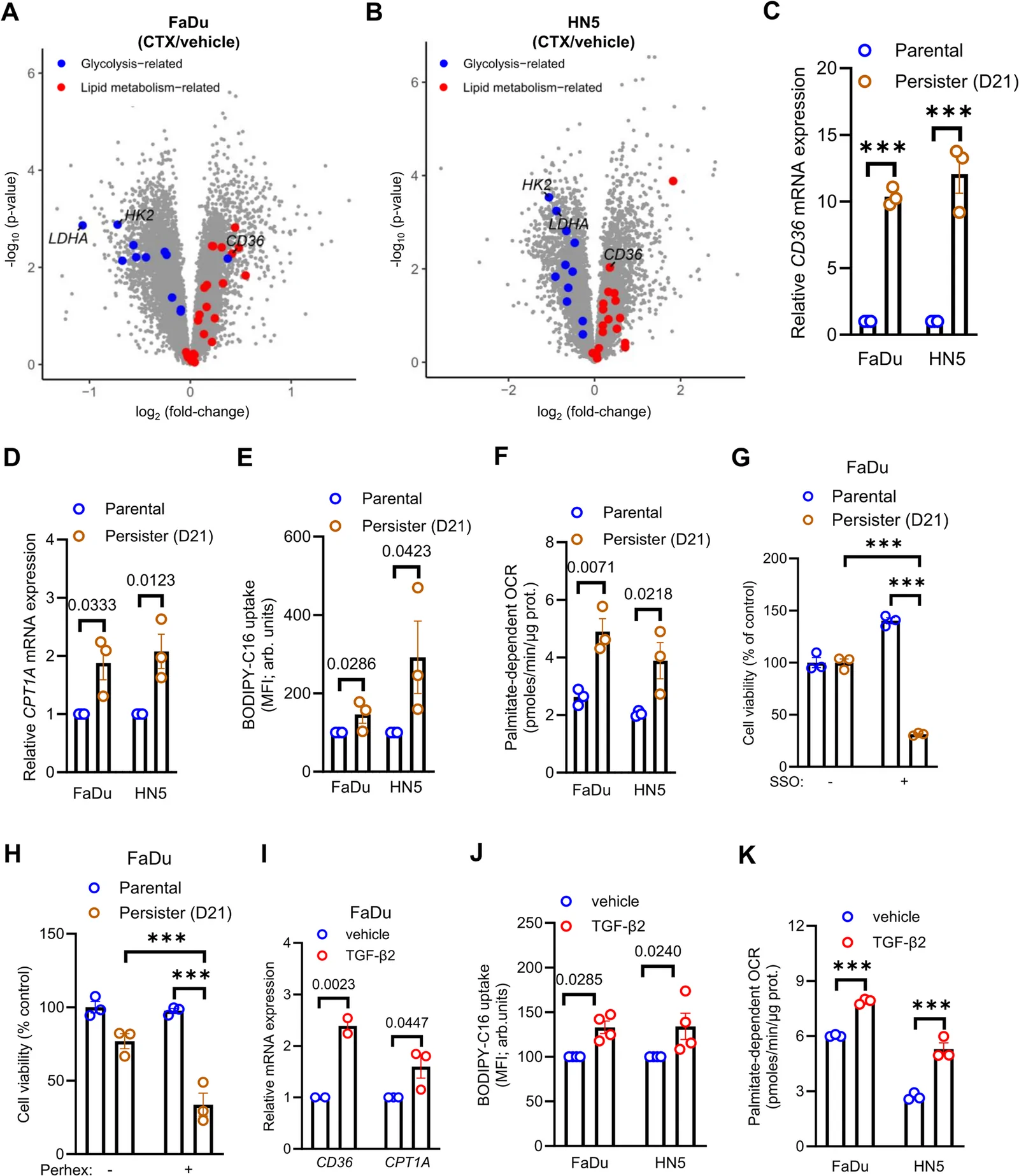
Residual HNSCC cells display TGFβ2-dependent enhanced fatty acid metabolism upon anti-EGFR therapy. **A-B** Volcano plots depicting an enrichment for lipid metabolism-related genes in FaDu **A** and HN5 **B** cells upon treatment with 1 µg/mL for 72 h (GSE306480) (*N* = 3). **C-F** mRNA expression for *CD36* (*N* = 3; *n* = 3) **C**, and *CPT1A* (*N* = 3; *n* = 3) **D**, BODIPY-C16 uptake (*N* = 3) **E**, and palmitate-dependent oxygen consumption rate (OCR) (*N* = 3; *n* = 6) **F** in FaDu and HN5 cells upon treatment with 1 µg/mL cetuximab for 21 days. **G-H** Viability of FaDu cells treated with 1 µg/mL cetuximab for 21 days, and then with 100 µM sulfo-N-succinimidyl oleate (SSO) **G**, and 4 µM perhexiline maleate **H** for 72 h (*N* = 3; *n* = 6). **I-K** mRNA expression for *CD36* and *CPT1A* (*N* = 2–3; *n* = 3) **I**, BODIPY-C16 uptake (*N* = 4) **J**, and palmitate-dependent OCR (*N* = 3; *n* = 6) **K** in FaDu and HN5 cells upon treatment with 10 ng/mL TGF-β2 for 48 h. Data are plotted as the means ± SEM **C-K**. *N* indicates the number of independent biological experiments and *n* indicates the number of technical replicates (when > 1). Significance was determined by two-way ANOVA with Tukey’s multiple comparison test **C-K**. *P*-values as indicated or ***P < 0.001

To explore the functional consequences of TGF-β2 induction, we analyzed transcriptional programs in cetuximab-treated tumor cells. RNA-sequencing revealed a consistent upregulation of lipid metabolism in persister HNSCC cells upon cetuximab treatment (Figs. 2A-B). In particular, CD36 and CPT1A, key regulators of FA uptake and oxidation, were significantly upregulated in persister HNSCC cells in vitro (Figs. 2C-D). Functionally, cetuximab-tolerant cells exhibited enhanced palmitate uptake and FA oxidation capacity (Figs. 2E-F and Supplementary Figure S2A), accompanied by lipid droplet accumulation (Supplementary Figure S2B). These metabolic changes conferred selective vulnerability to inhibitors targeting FA uptake (sulfo-N-succinimidyl oleate sodium (SSO)), oxidation (perhexiline maleate), or lipid storage (A922500) (Figs. 2G-H and Supplementary Figures S2C-F). Importantly, recombinant TGF-β2 recapitulated these metabolic changes by inducing *CD36* and *CPT1A* gene expression (Fig. 2I and Supplementary Figure S2G), and enhancing FA uptake and oxidation capacities (Figs. 2J-K), implicating TGF-β2 as a driver of this metabolic reprogramming.

### TGFβ2-reprogrammed CAFs confer paracrine resistance to EGFR blockade

**Fig. 3:**
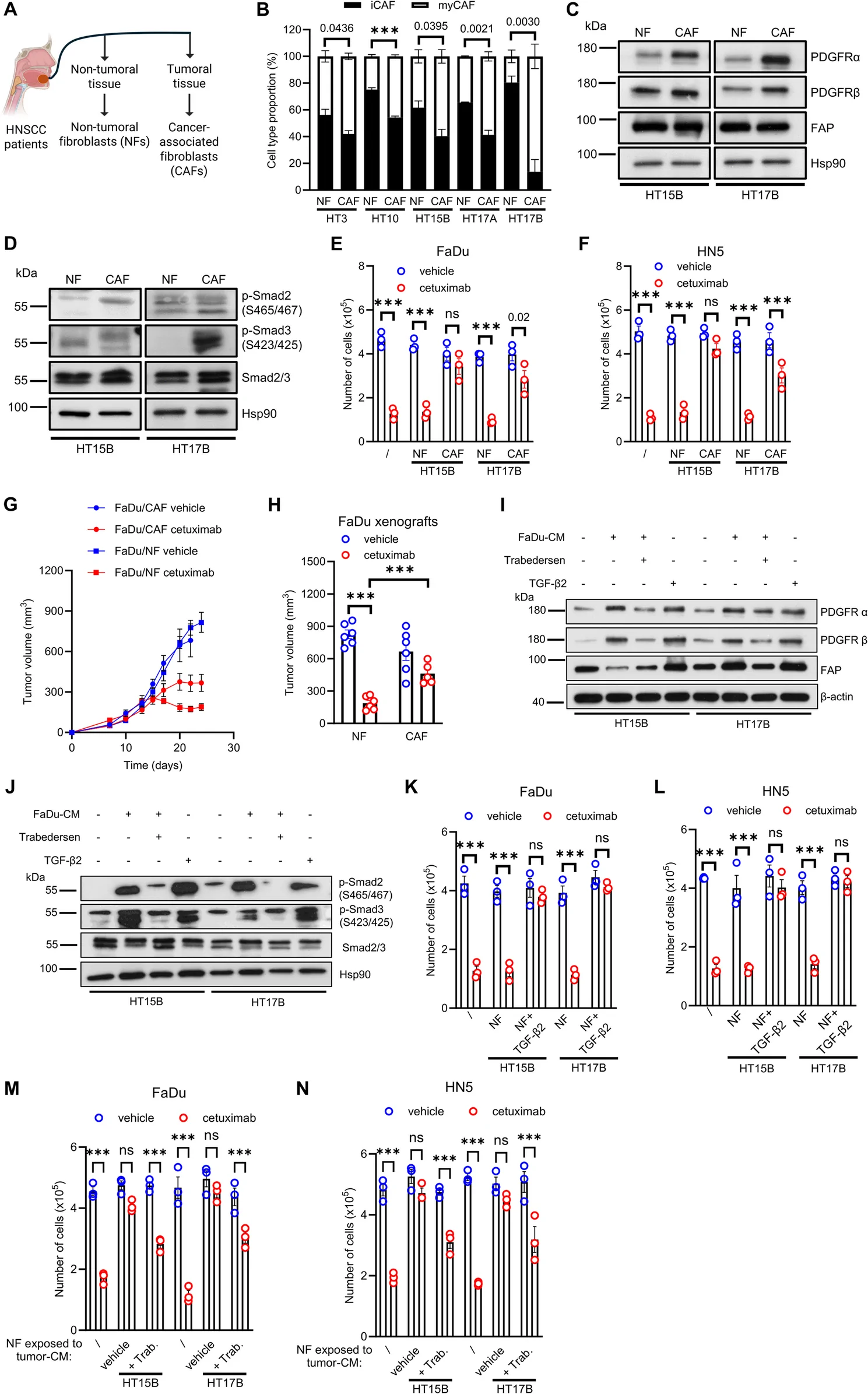
TGFβ2-activated myofibroblastic CAFs support anti-EGFR therapy resistance in HNSCC. **A** Experimental workflow for the establishment of matching non-tumoral and cancer-associated fibroblast lines from HNSCC patient-derived tissues. Created in BioRender. **B** Inferred proportion, after deconvolution of RNA-seq data (GSE303690), of inflammatory (iCAF) and myofibroblastic (myCAF) fibroblasts in HNSCC-derived CAF and NF cell populations (*N* = 3). **C-D** Representative immunoblots for platelet-derived growth factor receptor beta (PDGFRβ) alpha (PDGFRα) and fibroblast activation protein (FAP) **C**, and phosphorylated and total forms of Smad2/3 **D** in NF and CAF cell populations (HT15B and HT17B models) (*N* = 3). **E-F** Viability of FaDu **E** and HN5 **F** cells, co-cultured with NFs or CAFs (Transwell-based system) and treated with 1 µg/mL cetuximab for 72 h (*N* = 3; *n* = 6). **G-H** Growth follow-up **G** and volume at day 24 **H** of tumor xenografts from FaDu cells subcutaneously co-injected with NFs or CAFs (ratio 1:2 FaDu: fibroblasts) in nude mice, and then weekly treated with 30 mg/kg cetuximab (*N* = 5–6). **I-J** Representative immunoblots for platelet-derived growth factor receptor alpha (PDGFRα), beta (PDGFRβ), and fibroblast activation protein (FAP) **I**, and phosphorylated and total forms of Smad2/3 **J** in NF and CAF cell populations (HT15B and HT17B models) treated with 10 ng/mL TGF-β2 for 6 h or in presence of conditioned medium of FaDu with or without pre-incubation with 10 µM Trabedersen for 7 days. (*N* = 3). **K-L** Viability of FaDu **K** and HN5 **L** cells co-cultured with vehicle- or TGFβ2-treated NFs (Transwell-based system), and then treated with 1 µg/mL cetuximab for 72 h (*N* = 3; *n* = 6). **M-N** Viability of FaDu **M** and HN5 **N** cells co-cultured with NFs pre-exposed to CM from tumor cells treated or not with 10 µM Trabedersen for 7 days (Transwell-based system), and then treated with 1 µg/mL cetuximab for 72 h (*N* = 3; *n* = 6). Data are plotted as the means ± SEM **B**, **E-H**, and **K-N**. *N* indicates the number of independent biological experiments and *n* indicates the number of technical replicates (when > 1). Significance was determined by two-way ANOVA with Sidák’s multiple comparison test **B**,** E-F**,** H** and **K-N**. *P*-values as indicated or ***P < 0.001; ns, not significant

Given the dual autocrine and paracrine roles of TGF-β2, we next explored whether it modulates stromal fibroblasts to support cetuximab resistance. Primary CAFs and matched fibroblasts (from histologically normal cheek mucosa, collected at sites distant from the tumor) were isolated from HNSCC patients (Fig. 3A). Notably, given that all donors had a documented history of tobacco and/or alcohol exposure (Supplementary Figure S3A), these control fibroblasts were designated non-tumoral fibroblasts (NFs), rather than normal or healthy tissue fibroblasts. All cultures exhibited fibroblast-like morphology (Supplementary Figure S3B), and lineage purity was validated by flow cytometry using EpCAM, CD45, and CD31 markers to exclude contamination from epithelial cancer cells, immune cells, and endothelial cells, respectively (Supplementary Figure S3C). Following tissue dissociation, single-cell suspensions were expanded in fibroblast growth medium until cultures reached a proliferative state. We first performed bulk RNA sequencing on five matched CAF/NF pairs isolated from HNSCC patients. Principal component analysis revealed a clear transcriptional separation between CAF and NF populations (Supplementary Figure S3D), with about 3,000 genes being either up or downregulated in all CAF populations (vs. NF) (Supplementary Figure S3E). Deconvolution analysis using BayesPrism [36] and a single-cell CAF atlas [32] as prior information showed that both CAFs and NFs expressed high levels of genes associated with the immunosuppressive CAF-S1 subset [37] (Supplementary Figures S3F-H). Notably, CAFs exhibited significantly higher expression of myofibroblast-associated (myCAF) genes, as well as elevated PDGFR-α/β protein levels, compared to NFs (Figs. 3B-C and Supplementary Figure S3I). This myCAF subtype has been linked to elevated TGF-β signaling [38]. Consistent with this, we observed increased phosphorylation of Smad2/3 in CAFs (Fig. 3D).

Using an indirect Transwell co-culture model, we found that CAFs, but not matched NFs, rescued (at least partly) the viability of cetuximab-treated HNSCC cells (Figs. 3E-F). Similarly, co-injection of CAFs, but not NFs, with FaDu cells in vivo reduced the antitumor activity of cetuximab (Figs. 3G-H). Moreover, treating NFs with recombinant TGF-β2 or conditioned medium (CM) from cetuximab-treated HNSCC cells favored the transition towards a CAF-like phenotype, characterized by increased PDGFR-α/β expression and activation of Smad signaling (Figs. 3I-J). These reprogrammed fibroblasts subsequently acquire the capacity to protect HNSCC cells from EGFR blockade (Figs. 3K-N). Importantly, these effects were abrogated when *TGFB2* gene was repressed with Trabedersen (Figs. 3M-N**)**, confirming the paracrine role of tumor-derived TGF-β2.

### Myofibroblastic CAFs provide FA to support anti-EGFR therapy resistance in HNSCC cells

**Fig. 4:**
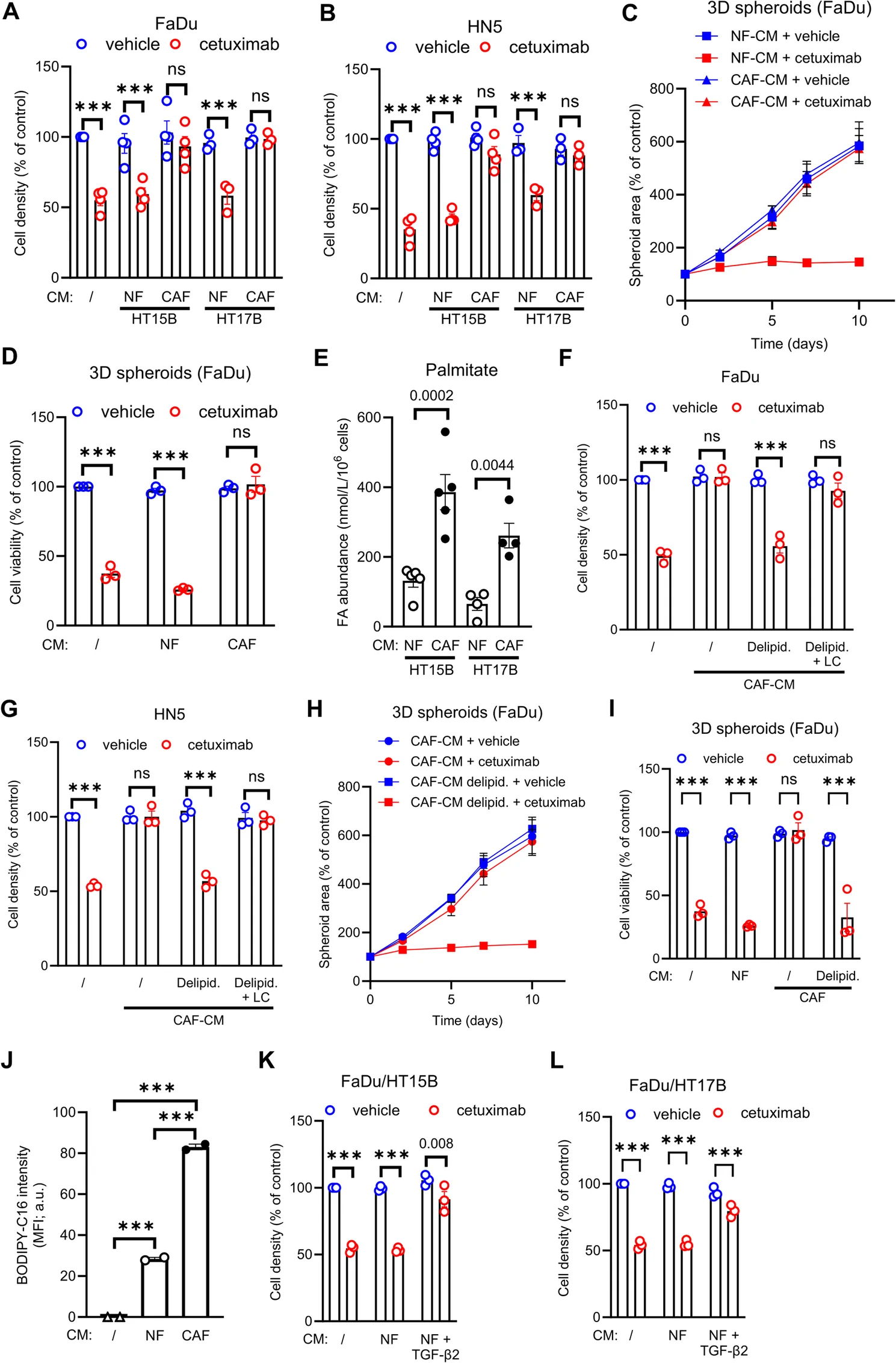
myCAFs act as FA providers to support anti-EGFR therapy resistance in HNSCC cells. **A-B** Viability of FaDu **A** and HN5 **B** cells after treatment with 1 µg/mL cetuximab for 72 h, in presence of conditioned medium (CM; 50% final volume) from NFs or CAFs (HT15B and HT17B models) (*N* = 3; *n* = 6). **C-D** Growth follow-up **C** and viability at day 10 **D** of FaDu spheroids after treatment with 1 µg/mL cetuximab for 10 days, in presence of conditioned medium (CM; 50% final volume) from NFs or CAFs (HT15B model) (*N* = 3; *n* = 6). **E** Abundance of palmitate in conditioned medium from NFs or CAFs (HT15B and HT17B models) (*N* = 4–5). **F-G** Viability of FaDu **F** and HN5 **G** cells after treatment with 1 µg/mL cetuximab for 72 h, in presence of CAF-CM (50% final volume; HT15B model) upon delipidation, with or without supplementation with chemically defined lipid concentrate (LC) (*N* = 3; *n* = 6). **H-I** Growth follow-up **H** and viability at day 10 **I** of FaDu spheroids after treatment with 1 µg/mL cetuximab for 10 days, in presence of CAF-CM (50% final volume; HT15B model) upon delipidation (*N* = 3; *n* = 6). **J** Fluorescence intensity in FaDu cells upon incubation for 6 h with conditioned medium from NFs or CAFs pre-loaded with BODIPY-C16 (*N* = 2). **K-L** Viability of FaDu cells after treatment with 1 µg/mL cetuximab for 72 h, in presence of conditioned medium (CM; 50% final volume) from NFs of HT15B **K** and HT17B **L** models pre-treated or not with 10 ng/mL TGF-β2 for 72 h (*N* = 3; *n* = 6). Data are plotted as the means ± SEM **A-L**. *N* indicates the number of independent biological experiments and *n* indicates the number of technical replicates (when > 1). Significance was determined by two-way ANOVA with Sidák’s multiple comparison test **A-B**, **D**, **F-G**, **I** and **K-L**, or one-way ANOVA with Dunnett’s multiple comparison test **E** and **J**. *P*-values as indicated or ***P < 0.001; ns, not significant

We next investigated whether myCAF-derived FA contribute to tumor cell survival during EGFR inhibition. CAF-derived CM, but not NF-derived CM, restored the viability of cetuximab-treated HNSCC cells in both 2D and 3D culture conditions (Figs. 4 A-D and Supplementary Figures S4A-E). Gas chromatography with flame ionization detection (GC-FID) analysis confirmed significantly higher FA levels, particularly palmitic acid (C16:0), in CAF-CM compared to NF-CM (Fig. 4E and Supplementary Figure S4F). Depleting lipids from CAF-CM with fumed silica eliminated its protective effect in both 2D and 3D culture conditions (Figs. 4 F-I and Supplementary Figures S4G-H). Importantly, this method did not alter protein levels for EGFR ligands, namely EGF and TGF-α, in CAF-CM (Supplementary Figures S4I-J). Addition of a chemically defined lipid concentrate (LC), consisting of a mixture of FA and cholesterol, to the lipid-depleted CAF-CM was sufficient to restore the protective effect against cetuximab (Figs. 4 F-G and Supplementary Figure S4H). We further demonstrated that CAFs more effectively transferred fluorescently labeled FA (BODIPY-C16) to HNSCC cells than NFs (Fig. 4J). Moreover, CM from TGFβ2-treated NF also protected HNSCC cells from cetuximab (Figs. 4K-L**).** Together, these data highlight a stromal metabolic axis wherein tumor-derived TGF-β2 drives FA secretion from myCAFs, promoting a drug-tolerant phenotype in HNSCC cells.

### myCAF-derived FA support bioenergetic adaptation and EGFR signaling in HNSCC cells

**Fig. 5:**
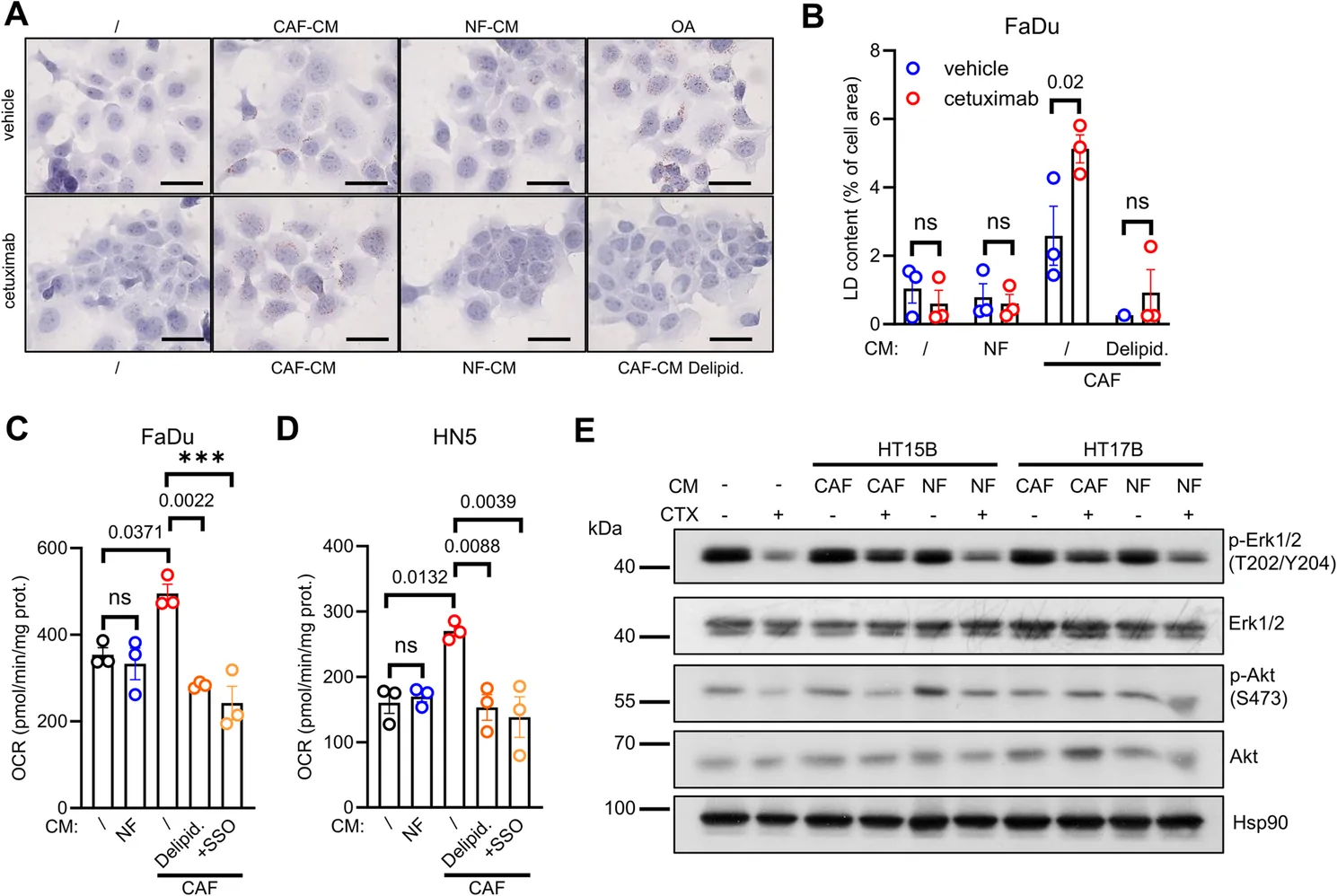
myCAF-derived FA support bioenergetic needs and EGFR signaling in HNSCC cells. **A-B** Representative brightfield pictures **A** and quantification **B** of ORO-stained LD in FaDu cells treated with 1 µg/mL cetuximab for 72 h in presence or not of (delipidated) conditioned medium from NFs and CAFs (HT15B model). Treatment with 50 µM BSA-conjugated oleic acid (OA) was used as positive control (*N* = 3). Scale bar: 100 μm. **C-D** Basal oxygen consumption rate (OCR) in FaDu **C** and HN5 **D** cells treated with (delipidated) conditioned medium from NFs and CAFs, in presence or not of 10 µM SSO (*N* = 3; *n* = 6). **E** Representative immunoblots for phosphorylated and total forms of Erk1/2 and Akt in FaDu cells treated with 1 µg/mL cetuximab for 72 h in presence of conditioned medium of NFs and CAFs (HT15B and HT17B models) (*N* = 3). Data are plotted as the means ± SEM **B-D**. *N* indicates the number of independent biological experiments and *n* indicates the number of technical replicates (when > 1). Significance was determined by two-way ANOVA with Sidák’s multiple comparison test **B**, or one-way ANOVA with Dunnett’s multiple comparison test **C-D**. *P*-values as indicated or ***P < 0.001; ns, not significant

We next characterized the effects of CAF-derived FA on HNSCC cells exposed to cetuximab. Oil Red O staining revealed increased lipid droplet accumulation in HNSCC cells cultured with CAF-CM (Figs. 5 A-B and Supplementary Figures S5A-D), indicating enhanced intracellular lipid storage. Seahorse analysis performed after 72 h of incubation with CAF-CM or NF-CM revealed significantly higher oxygen consumption rates (OCR) in cells exposed to CAF-CM than in cells cultured with NF-CM or serum-free control medium (Figs. 5C-D and Supplementary Figure S5E). This increase in mitochondrial respiration was abolished either by delipidation of CAF-CM or by pre-treatment of HNSCC cells with SSO (Figs. 5C-D and Supplementary Figure S5E). CAF-CM, but not NF-CM, also sustained MAPK signaling, as evidenced by persistent Erk1/2 phosphorylation in cetuximab-treated HNSCC cells (Fig. 5E). Together, these data support a model wherein CAF-derived FA are imported by HNSCC cells to fuel oxidative metabolism and sustain EGFR/MAPK signaling, thereby promoting cetuximab tolerance.

### Therapeutic targeting of tumor/stroma metabolic crosstalk impairs cetuximab resistance

**Fig. 6:**
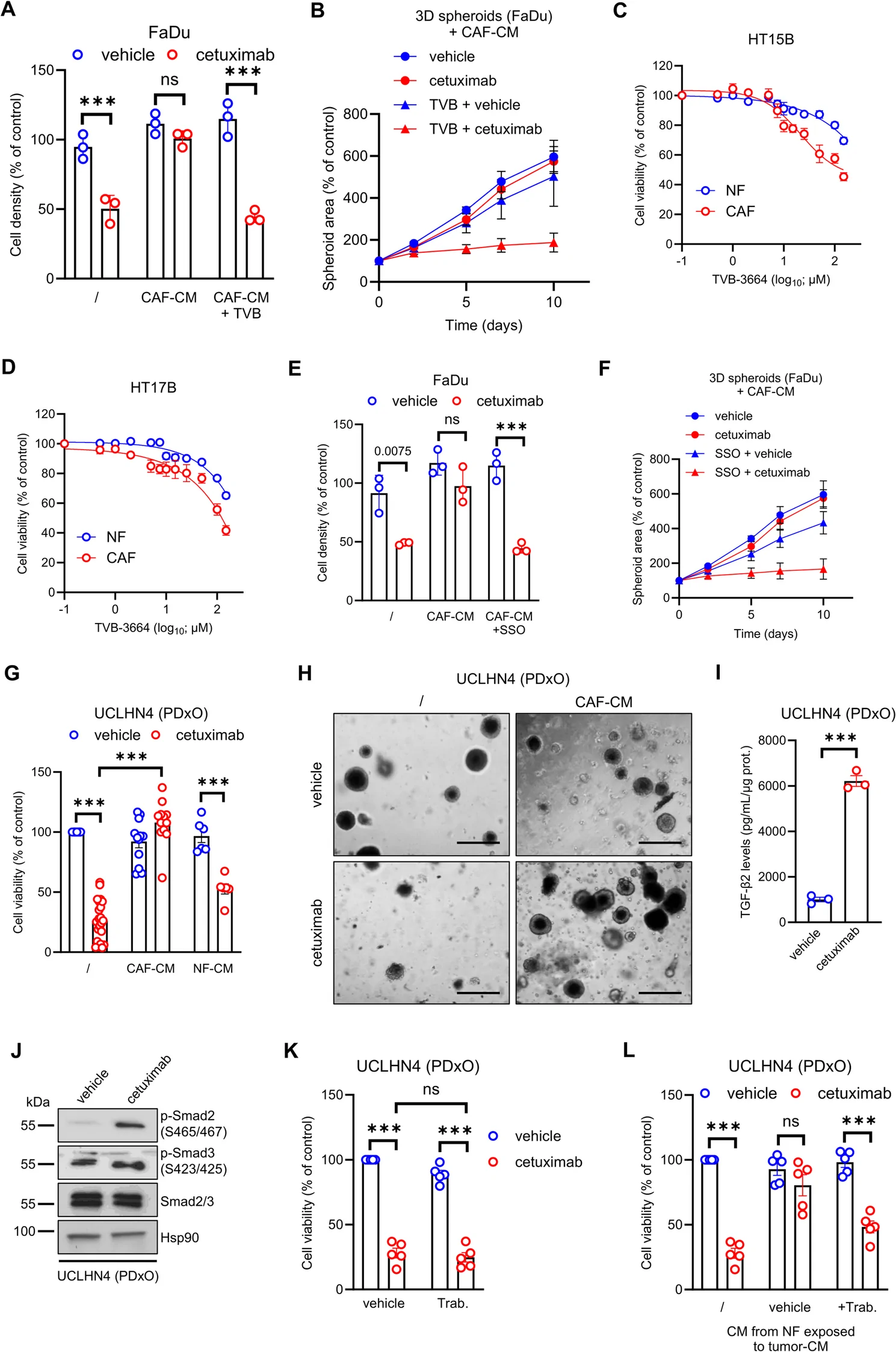
Therapeutic targeting of tumor/stroma metabolic crosstalk impairs cetuximab resistance in HNSCC cells. **A-B** Viability of FaDu cells in 2D **A** and 3D **B** conditions upon treatment with 1 µg/mL cetuximab in presence or not of conditioned medium from CAFs (HT15B model) pre-treated or not with 10 µM TVB-3664 for 72 h (*N* = 3; *n* = 6). **C-D** Viability of NFs and CAFs from HT15B **C** and HT17B **D** models upon treatment with increasing concentrations of TVB-3664 for 72 h (*N* = 3; *n* = 6). **E-F** Viability of FaDu cells in 2D **E** and 3D **F** conditions upon treatment with 1 µg/mL cetuximab in presence or not of conditioned medium from CAFs (HT15B model) and 100 µM SSO (*N* = 3; *n* = 6). **G-H** Viability **G** and representative brightfield pictures **H** of PDX-derived HNSCC organoids (UCLHN4 model) upon treatment with 2 µg/mL cetuximab in presence or not of CAF-CM (HT15B model) for 2 weeks (*N* = 6). Scale bar: 200 μm. **I-J** Active secreted TGF-β2 protein levels **I** and representative immunoblot for phosphorylated and total forms of Smad2/ **J** in PDX-derived HNSCC organoids (UCLHN4 model) upon treatment with 2 µg/mL cetuximab for 2 weeks (*N* = 3). **K-L** Viability of PDX-derived HNSCC organoids (UCLHN4 model) upon treatment for 2 weeks with 2 µg/mL cetuximab in presence or absence of 10 µM Trabedersen **K** or conditioned medium from NF pre-exposed to CM from Trabedersen-treated FaDu cells **L**. Data are plotted as the means ± SEM **A-G**,** I** and **K-L**. *N* indicates the number of independent biological experiments and *n* indicates the number of technical replicates (when > 1). Significance was determined by two-way ANOVA with Sidák’s multiple comparison test **A**, **E**, **G**, and **K-L**, or two-tailed unpaired Student’s t-test **I**. *P*-values as indicated or ***P < 0.001; ns, not significant

To determine whether this signaling-metabolic crosstalk could be therapeutically exploited, CAFs were treated with TVB-3664, a pharmacological inhibitor of fatty acid synthase (FASN). TVB-3664 significantly reduced FA levels in CAF-CM, as measured by GC-FID (Supplementary Fig. 6A). This FA-depleted CAF-CM was less effective at mitigating cetuximab-induced growth inhibition in HNSCC cells in both 2D and 3D models (Figs. 6A-B and Supplementary Figures S6B-C), suggesting that CAF-derived FA contribute directly to the protective effect. Notably, CAFs were more sensitive to FASN inhibition than NFs (Figs. 6C-D). Likewise, inhibition of FA uptake in HNSCC cells with SSO abolished the protective effect of CAF-CM against cetuximab in both monolayer cultures and spheroids (Figs. 6E-F and Supplementary Figures S6D-E). In patient-derived organoids (PDOs), cetuximab significantly increased cytotoxicity (Supplementary Figure S6F), whereas CAF-CM protected organoids from cetuximab-induced growth inhibition in a lipid-dependent manner (Figs. 6G-H and Supplementary Figure S6G). Importantly, cetuximab treatment increased active TGF-β2 levels in PDO-derived CM (Fig. 6I), together with enhanced Smad signaling (Fig. 6J). These findings identify FA synthesis and uptake as actionable vulnerabilities capable of disrupting the tumor-CAF metabolic axis and restoring cetuximab sensitivity.

To evaluate the therapeutic relevance of TGF-β2 inhibition, we performed additional experiments using Trabedersen in PDO models. Cetuximab significantly reduced organoid viability, whereas Trabedersen alone had no detectable effect (Fig. 6K). Moreover, combining Trabedersen with cetuximab did not further decrease viability compared with cetuximab alone. These results indicate that direct TGF-β2 inhibition does not enhance the short-term cytotoxic effects of cetuximab in PDO monocultures. We next investigated whether TGF-β2 inhibition could disrupt tumor-stroma communication. NFs were exposed to CM collected from cetuximab-treated HNSCC cells in the presence or absence of Trabedersen, and CM from these “educated” fibroblasts was subsequently transferred to cetuximab-treated PDOs. CM from NFs exposed to tumor-derived CM protected organoids against cetuximab-induced growth inhibition (Fig. 6L). In contrast, when the initial tumor CM was generated from Trabedersen-treated HNSCC cells, the resulting NF-derived CM no longer conferred protection. These findings indicate that TGFβ2-dependent stromal reprogramming contributes to cetuximab tolerance.

### TGFβ2-dependent tumor-stroma metabolic crosstalk correlates with cetuximab tolerance in patient-derived HNSCC models

**Fig. 7:**
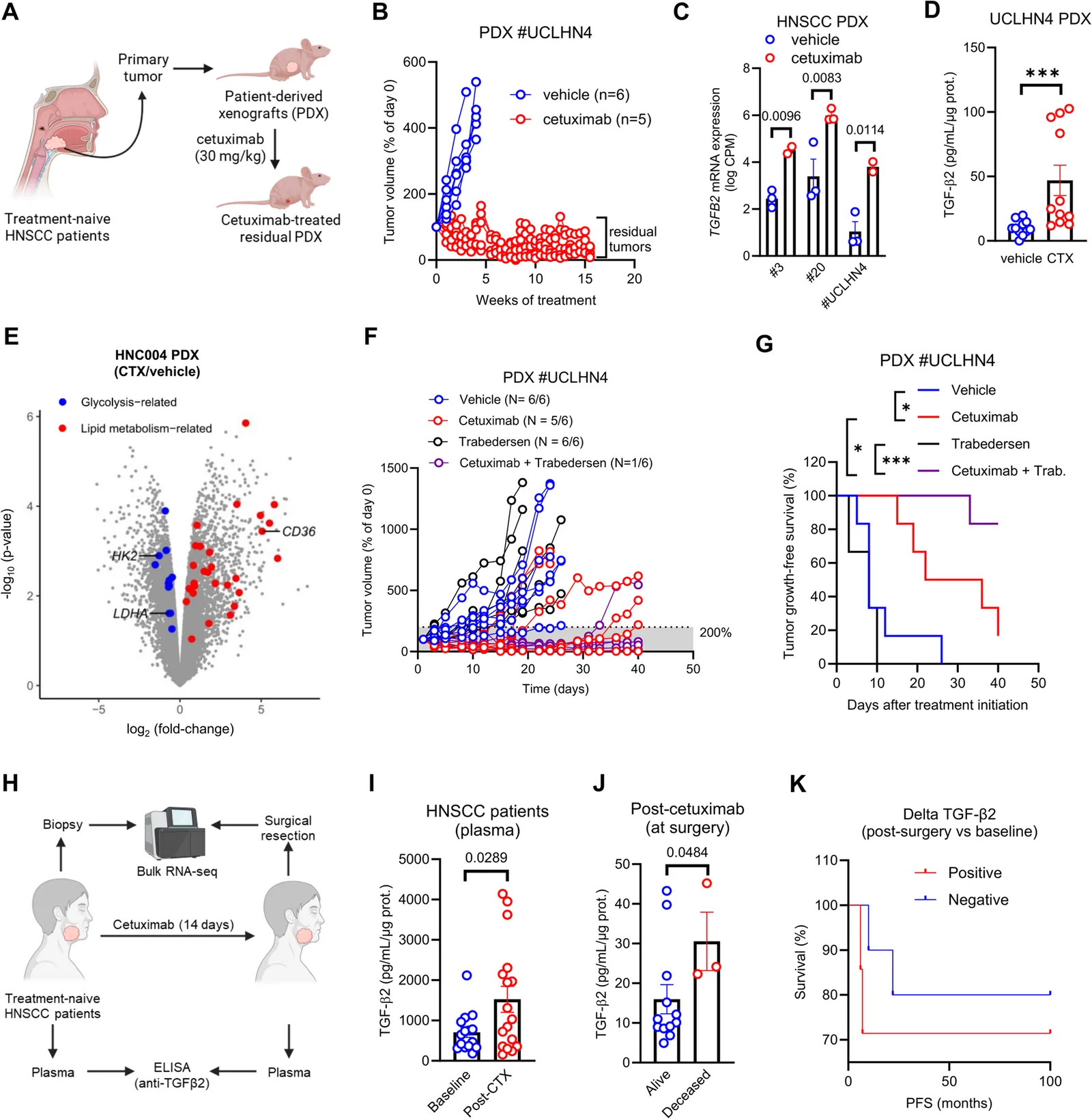
TGFβ2-dependent tumor-stroma metabolic crosstalk correlates with cetuximab tolerance in patient-derived HNSCC models. **A** Schematic representation for the establishment and treatment of patient-derived xenograft (PDX) models from treatment-naive HNSCC clinical specimens. Created in BioRender. **B** Tumor growth of cetuximab-sensitive UCLHN4 PDX in nude mice weekly treated with 30 mg/kg cetuximab or vehicle (*N* = 5–6). Xenografts were considered residual tumors when the size stabilized for at least 4 weeks under cetuximab treatment. **C** Expression levels of TGF-β2 mRNA in cetuximab-treated UCLHN4, #03 and #20 PDX models (*N* = 3) (GSE303798 and GSE143279). **D** Active TGF-β2 protein levels in cell lysates from vehicle- and cetuximab-treated UCLHN4 PDX (*N* = 11). **E** Volcano plot depicting an enrichment for lipid metabolism-related genes in cetuximab-treated HNC004 PDX model (*N* = 3). **F-G** Growth follow-up of individual tumor xenografts **F** and Kaplan-Meier survival curves **G** from UCLHN4 PDX in nude mice treated with 30 mg/kg cetuximab, 50 mg/kg Trabedersen (for the first dose and then 16 mg/kg), alone or in combination (*N* = 6). In G, events were defined as the first time tumor volume reached ≥ 200% of baseline (day 0) tumor volume. Mice not reaching the threshold during follow-up were censored at their last measurement. **H** Schematic representation for the clinical workflow of the window-of-opportunity study with HNSCC patients treated for 14 days with cetuximab. Tissue and plasma samples were collected before and after cetuximab treatment. Created in BioRender. **I** TGF-β2 levels in plasma from HNSCC patients, before (baseline) and after treatment with cetuximab for 14 days (*N* = 12). **J** Post-cetuximab TGF-β2 levels in plasma from HNSCC patients (alive or deceased after surgery) (*N* = 15). **K** Kaplan-Meier survival plot of PFS for HNSCC patients according to the variation of TGF-β2 levels in plasma before and after cetuximab treatment. Data are plotted as the means ± SEM **C-D** and **I-K**. *N* indicates the number of independent biological experiments and *n* indicates the number of technical replicates (when > 1). Significance was determined by two-tailed unpaired Student’s t-test **D**,** I**, or two-tailed paired Student’s t-test **J**. In **C**, adjusted p-values determined from RNA-sequencing data analysis are indicated in the graph. *P*-values as indicated

To assess the clinical relevance of our findings, we analyzed residual tumors from cetuximab-treated HNSCC patient-derived xenografts (PDX). Residual disease was defined as tumors exhibiting stable size for at least four weeks after treatment initiation, corresponding to maximal response (Figs. 7 A-B). Transcriptomic analysis revealed that approximately 20% of sequencing reads originated from human tumor cells, whereas the remaining 80% derived from murine stromal cells (Supplementary Figure S7A). This stromal composition closely mirrors the TME observed in advanced-stage HNSCC patients [17]. We further analyzed publicly available RNA-sequencing datasets from two additional cetuximab-sensitive HNSCC PDX models (#3 and #20) [39]. In all cases, *TGFB2*, but not *TGFB1* and *TGFB3*, was specifically upregulated following cetuximab treatment (Fig. 7C and Supplementary Figures S7B-C). Consistent with these transcriptomic findings, ELISA analyses on tumor lysates demonstrated significantly increased TGF-β2 protein levels in cetuximab-treated tumors (Fig. 7D), confirming that TGF-β2 induction occurs at both the transcript and protein levels during the early adaptive response to cetuximab in vivo. In PDX models with acquired cetuximab resistance, *TGFB2*, but not *TGFB1* and *TGFB3* genes, was likewise consistently upregulated (Supplementary Figure S7D). In parallel, genes involved in FA metabolism, including *CD36* and *CPT1A*, were upregulated in residual cetuximab-treated tumors (Fig. 7E and Supplementary Figures S7E-F). To determine whether TGF-β2 blockade could improve cetuximab efficacy in vivo, we treated UCLHN4 PDX-bearing mice with cetuximab alone or in combination with Trabedersen. Trabedersen monotherapy had no significant effect on tumor growth (Figs. 7F-G). As expected, cetuximab induced an initial tumor regression followed by progressive regrowth after several weeks, with five of six tumors ultimately exceeding 200% of their initial volume despite continued treatment. Importantly, combining cetuximab with Trabedersen significantly delayed tumor regrowth, with only one mouse exhibiting tumor progression during the observation period (Figs. 7F-G). No overt toxicity was observed in any treatment group, as assessed by longitudinal monitoring of mouse body weight (Supplementary Figure S7G). Together, these findings support the therapeutic potential of combining TGF-β2 inhibition with cetuximab.

Finally, to investigate the clinical relevance of TGFβ2-associated metabolic reprogramming, we analyzed RNA-sequencing and plasma samples from a previously reported window-of-opportunity study in which treatment-naive HNSCC patients received cetuximab for 14 days (Fig. 7H) [40]. Transcriptomic analyses revealed enrichment of OXPHOS-related gene signatures following treatment (Supplementary Figure S7H). In parallel, circulating TGF-β2 levels were significantly increased in post-treatment plasma samples (Fig. 7I). Notably, post-treatment TGF-β2 levels, but not baseline levels, were significantly higher in deceased patients than in surviving patients, and increases in TGF-β2 levels following treatment were associated with shorter progression-free survival (Figs. 7J-K and Supplementary Figure S7I). Altogether, these clinical data indicate that activation of a TGF-β2-associated metabolic program is linked to reduced sensitivity to anti-EGFR therapy and poorer clinical outcomes in patients with HNSCC.

## Discussion

Despite the centrality of EGFR signaling in HNSCC, therapeutic strategies targeting this pathway, including cetuximab, continue to yield modest and often transient clinical benefits. While mechanisms of acquired resistance are well documented, the early adaptive processes enabling tumor persistence during treatment remain less well defined. Understanding these mechanisms is critical for improving the depth and durability of response. Here, we integrate previously disconnected observations, including lipid metabolism reprogramming, TGF-β signaling, and fibroblast activation, into a unified mechanistic framework. We demonstrate that TGF-β2 drives a reciprocal tumor-stroma metabolic circuit that sustains a reversible drug-tolerant state during EGFR blockade.

Non-proliferative but metabolically active persister cells contribute to minimal residual disease across multiple cancer types [41–45]. In HNSCC, therapy-tolerant states have been linked to mesenchymal plasticity and ferroptosis sensitivity [46, 47]. Our findings identify TGF-β2 as a central mediator of early cetuximab tolerance, characterized by activation of canonical Smad signaling and increased dependence on FA metabolism. Importantly, TGF-β2 induction was consistently observed across multiple experimental systems, including HNSCC cell lines, patient-derived organoids, and patient-derived xenografts, and was associated with elevated circulating TGF-β2 levels in cetuximab-treated patients. These observations extend previous reports linking cetuximab resistance to epithelial-mesenchymal transition (EMT)-like reprogramming and enhanced TGF-β signaling [48] by identifying a specific and functionally relevant role for the TGF-β2 isoform.

TGF-β signaling regulates numerous aspects of cancer biology including immune evasion, fibrosis, and dormancy [49–55]. Among the three TGF-β isoforms, TGF-β2 has emerged as a key regulator of therapy-induced cellular plasticity and metabolic adaptation [56–59]. Consistent with these reports, we found that cetuximab-induced TGF-β2 promotes a metabolic shift toward FA uptake, oxidation, and storage, thereby creating a state of increased metabolic flexibility. Notably, inhibition of TGF-β2 preferentially impaired cetuximab-tolerant cells while having limited effects on untreated parental cells, supporting a context-dependent role for TGF-β2 during adaptive drug tolerance. Although immune-mediated mechanisms were not directly examined in the present study, our findings complement previous work demonstrating that TGF-β signaling can suppress cetuximab-mediated immune cytotoxicity and contribute to therapeutic resistance in vivo [60]. Future studies incorporating immune-competent models will be valuable to determine how metabolic, stromal, and immune functions of TGF-β2 cooperate during cetuximab treatment.

Mechanistically, we demonstrate that autocrine TGF-β2 signaling enhances FA transport, oxidation, and storage in HNSCC cells exposed to EGFR blockade. This FA-dependent phenotype resembles that reported in EGFR-inhibited drug-tolerant cells from NSCLC, where survival relies on FAO-driven oxidative metabolism [61, 62]. Accordingly, cetuximab-treated HNSCC cells displayed increased vulnerability to inhibitors of FA uptake, oxidation, and lipid storage, highlighting a metabolic liability associated with adaptive resistance.

Beyond its tumor cell-intrisic effects, TGF-β2 also acted as a potent stromal reprogramming factor. We show that tumor-derived TGF-β2 converts non-tumoral fibroblasts into CAF-like myofibroblasts characterized by increased Smad activation, elevated PDGFR expression, and enhanced lipid secretion. These myCAFs supplied FA to tumor cells, thereby sustaining mitochondrial respiration and maintaining MAPK signaling despite EGFR blockade. These findings align with reports showing that CAFs supply nutrients such as alanine, lactate, and glutamine to support tumor growth, and parallel lipid-based crosstalk observed in colorectal cancer [63], PDAC [64], and TKI-resistant NSCLC [65, 66]. Moreover, NSD1-dependent regulation of the TGFβ2/PGC1α axis in HNSCC influences mitochondrial respiration and metabolic inhibitor sensitivity [67]. Our work extends these observations by identifying TGF-β2 as the molecular signal linking EGFR inhibition in tumor cells to stromal metabolic reprogramming. Furthermore, the observation that conditioned medium from fibroblasts exposed to TGF-β2-rich tumor secretomes protected patient-derived organoids from cetuximab, whereas this protection was lost when TGF-β2 production was blocked, directly demonstrates the functional importance of this tumor-stroma communication axis.

The translational relevance of these findings is supported by experiments performed in clinically relevant PDO and PDX models. While Trabedersen alone did not affect organoid viability or PDX tumor growth, inhibition of TGF-β2 disrupted stromal-mediated protection and significantly delayed tumor regrowth following cetuximab treatment in vivo. Importantly, the lack of additional cytotoxicity observed in PDO monocultures suggests that the therapeutic value of TGF-β2 inhibition resides primarily in its ability to interfere with adaptive tumor-stroma interactions rather than directly killing tumor cells. These results reinforce the concept that adaptive resistance is an ecosystem-level process involving reciprocal signaling between tumor and stromal compartments.

Clinically, elevated TGF-β2 expression correlates with poor outcomes across malignancies and has prompted therapeutic targeting strategies [68–73]. Our analyses of cetuximab-treated HNSCC patients revealed increased circulating TGF-β2 levels following treatment, with higher post-treatment levels and larger treatment-induced increases associated with inferior clinical outcomes. Together with our preclinical findings, these observations support TGF-β2 as both a potential biomarker of adaptive response and a therapeutic target. More broadly, our study provides mechanistic support for combining EGFR inhibitors with TGF-β or lipid metabolism-targeting approaches to delay or prevent the emergence of drug tolerance [74–76].

## Conclusions

This study identifies a previously unrecognized mechanism of adaptive cetuximab tolerance in HNSCC in which tumor-derived TGF-β2 orchestrates a dual autocrine-paracrine program that enables tumor cell persistence under EGFR blockade. Autocrine TGF-β2 signaling reprograms tumor metabolism toward increased FA uptake, oxidation, and storage, creating a metabolically adaptable drug-tolerant state. In parallel, tumor-secreted TGF-β2 remodels the stromal microenvironment by converting fibroblasts into lipid-secreting myofibroblasts that supply exogenous FA necessary to sustain oxidative metabolism and maintain EGFR/MAPK signaling in tumor cells. Importantly, our study demonstrates in both PDO and PDX models that therapeutic inhibition of TGF-β2 disrupts this adaptive tumor-stroma communication network and enhances the durability of cetuximab response. Rather than exerting direct cytotoxic effects on tumor cells, TGF-β2 blockade primarily interferes with stromal reprogramming and metabolic support mechanisms that underlie adaptive resistance. These findings position TGF-β2 as a central driver of early drug tolerance and highlight tumor-stroma metabolic cooperation as a critical determinant of EGFR inhibitor response. While immune-mediated mechanisms were beyond the scope of the current study, our work complements prior evidence linking TGF-β2 to immune evasion and provides a framework for combining EGFR-targeted therapies with strategies aimed at disrupting TGF-β2 signaling or stromal lipid metabolism to improve treatment efficacy in HNSCC and potentially other stroma-rich epithelial malignancies.

## Supplementary Information

Supplementary Material 1.

Supplementary Material 2.

## Data Availability

Datasets from RNA-sequencing analyses, generated and/or used during this study, are available at Gene Expression Omnibus: GSE306480 (cetuximab-treated HNSCC cell lines; reviewer token: qvwnagksrzanzuh), GSE303690 (HNSCC patient-derived CAFs and NF; reviewer token: wngnoekmbpsfbsx), GSE303798 (cetuximab-treated residual UCLHN4 and HNC004 HNSCC PDX; reviewer token: knyvukkcrrwjvcz), GSE143279 (cetuximab-treated #03 and #020 HNSCC PDX), GSE235417 and GSE307896 (cetuximab-sensitive and -resistant HNSCC PDX; reviewer token: yfmtgyemfrgjxst). The tiling array expression dataset from cetuximab-treated HNSCC patients is also available at Gene Expression Omnibus: GSE109756. All unique reagents generated in this study will be made available on request to Prof. Cyril Corbet (cyril.corbet@uclouvain.be), with a completed material transfer agreement (MTA).

## Abbreviations

CAF: Cancer-associated fibroblast
CM: Conditioned medium
EGFR: Epidermal growth factor receptor
FA: Fatty acid
FDA: Food and drug administration
HNSCC: Head and neck squamous cell carcinoma
HPV: Human papillomavirus
LC: Lipid concentrate
MAPK: Mitogen-activated protein kinase
NF: Non-tumoral fibroblast
PDO: Patient-derived organoid
PDX: Patient-derived xenograft
TGF-β: Transforming growth factorβ
TME: Tumor microenvironment
