# Supplementary material for: Reciprocal signaling-metabolic crosstalk between fibroblasts and tumor cells drives cetuximab tolerance in head and neck cancers
Source: J Exp Clin Cancer Res. 2026 Jun 24;45:193. doi: 10.1186/s13046-026-03765-9 (PMC13551799; doi:10.1186/s13046-026-03765-9)
Supplement: Supplementary file 1 [file 13046_2026_3765_MOESM1_ESM.docx]

**Supplementary information**

**Reciprocal signaling-metabolic crosstalk between fibroblasts and tumor cells drives cetuximab tolerance in head and neck cancers**

Engy Vigneron, Julie Vignau, Olivier Lowyck, Valentin Van den bossche, Jérôme Ambroise, Yann Kieffer, Fatima Mechta-Grigoriou, Isabella Rizzi, Maria Virginia Giolito, Manon Desgres, Hannah Zaryouh, An Wouters, Antonella Mendola, Hajar Dahou, Marine Leclerc, Romain Boidot, Aurélien Warnant, Yvan Larondelle, Olivier Feron, Jean-Pascal Machiels, Sandra Schmitz, and Cyril Corbet

**Supplementary Figure 1. Residual HNSCC cells upregulate TGF-β2 under anti-EGFR therapy.** (**A-C**) Venn diagrams (**A**) and volcano plots (**B-C**) of differentially expressed genes in RNA-seq data (GSE306480) from FaDu and HN5 cells after treatment with 1 µg/mL cetuximab for 72 h (*N*=3). (**D**) mRNA expression levels of TGF-β2 in SC263 cells treated with 1 µg/mL cetuximab for 7, 14, and 21 days (*N*=3; *n*=3). (**E**) Representative immunoblots for phosphorylated and total forms of Smad3 in SC263 cells treated with 1 µg/mL cetuximab for 21 days or with 10 ng/mL TGF-β2 for 6 h (*N*=3). (**F**) Follow-up of viable cell number for HN5 cells treated with 1 µg/mL cetuximab up to 21 days. (**G**) Viability of parental and cetuximab-treated persister FaDu cells after treatment with 1 µg/mL cetuximab for 96 h (*N*=3). Persister cells were obtained after initial treatment with 1 µg/ml cetuximab for 21 days, and then followed or not with drug withdrawal for 7 days (reversal (rev.) condition). (**H**) Viability of SC263 cells after treatment with 1 µg/mL cetuximab for 7, 14, or 21 days, and then incubation with 10 µM Trabedersen for 7 days (*N*=3; *n*=6). (**I**) Viability of SC263 cells after treatment with 10 ng/mL TGF-β2 for 48h and then incubation with 1 µg/mL cetuximab for 96h (*N*=3; *n*=6). (**J-K**) mRNA expression (**J**) and active protein levels (**K**) for TGF-β2 in cetuximab-sensitive (-S) and -resistant (-R) HNSCC cells (*N*=3; *n*=3). (**L**) Representative immunoblots for phosphorylated and total forms of Smad2/3 in cetuximab-sensitive (-S) and -resistant (-R) FaDu cells (*N*=3). (**M**) mRNA expression levels of TGF-β2 in SC263 cells treated with 1 µg/mL cetuximab and 10 µM GW6471 for 72 h (*N*=3; *n*=3). Data are plotted as the means ± SEM (**D**, **F-K,** and **M**). *N* indicates the number of independent biological experiments and *n* indicates the number of technical replicates (when >1). Significance was determined by one-way ANOVA with Dunnett’s multiple comparison test (**D** and **M**), or two-way ANOVA with Sidák’s multiple comparison test (**H-K**). *P*-values as indicated or ****P*<0.001; ns, not significant.

**Supplementary Figure 2. Residual HNSCC cells display TGFβ2-dependent enhanced fatty acid metabolism upon anti-EGFR therapy.** (**A**) Gating strategy for flow cytometry analysis of BODIPY-C16 detection (FITC channel) in live single cells (FaDu and HN5 cells, see Fig. 2E). (**B**) Lipid droplet content in FaDu cells treated or not with 1 µg/mL cetuximab for 21 days (*N*=6). (**C-D)** Viability of HN5 cells treated with 1 µg/mL cetuximab for 21 days, and then with 100 µM sulfo-N-succinimidyl oleate (SSO) (**C**) or 4 µM perhexiline maleate (**D**) for 96h (*N*=3; *n*=6). (**E-F**) Viability of FaDu (**E**) and HN5 (**F**) cells treated with 1 µg/mL cetuximab for 21 days, and then with 40 µM A922500 for 72h (*N*=3; *n*=6). (**G**) mRNA expression for *CD36* and *CPT1A* in HN5 cells upon treatment with 10 ng/mL TGF-β2 for 48h (*N*=3; *n*=3). Data are plotted as the means ± SEM (**B-G**). *N* indicates the number of independent biological experiments and *n* indicates the number of technical replicates (when >1). Significance was determined by two-tailed unpaired Student’s t-test (**B**), and two-way ANOVA with Sidák’s multiple comparison test (**C-G**). *P*-values as indicated or ****P*<0.001; ns, not significant.

**Supplementary Figure 3. TGFβ2-activated myofibroblastic CAFs support anti-EGFR therapy resistance in HNSCC.** (**A**) Clinical data for tissue donor HNSCC patients from whom CAF and NF populations were derived. (**B**) Representative brightfield images of CAFs and NFs (HT15B and HT17B models). Scale bar: 100 µm. (**C**) Gating strategy for flow cytometry analysis of CD31, CD45, CD90, and EpCAM detection in live single cells (NFs and CAFs). (**D-E**) Principal component analysis plot (**D**) and volcano plot of differentially expressed genes (**E**) in RNA-seq data (GSE303690) from HNSCC patient-derived NFs and CAFs (*N*=3). (**F-H**) Expression levels for *ANTXR1* (**F**), *FAP* (**G**), and *SDC1* (**H**) genes in HNSCC-derived NFs and CAFs (*N*=3). (**I**) Inferred proportion, after RNA-seq deconvolution, of gene expression-based fibroblast subpopulations in HNSCC-derived CAF and NF cell populations (*N*=3). Data are plotted as the means ± SEM (**F-I**). *N* indicates the number of independent biological experiments and *n* indicates the number of technical replicates (when >1). Significance was determined by two-tailed unpaired Student’s t-test (**F-H**). *P*-values as indicated or ****P*<0.001.

**Supplementary Figure 4. myCAFs act as FA providers to support anti-EGFR therapy resistance in HNSCC.** (**A-B**) Viability of SC263 cells after treatment with 1 µg/mL cetuximab for 72h, in presence of conditioned medium (CM; 50% final volume) from NFs or CAFs; HT15B (**A**) and HT17B (**B**) models (*N*=3; *n*=6). (**C-E**) Viability of FaDu (**C**), HN5 (**D**), and SC263 cells (**E**) after treatment with 1 µg/mL cetuximab for 72h, in presence of conditioned medium (CM; 50% final volume) from NFs or CAFs (HT3, HT10, and HT17A models) (*N*=3; *n*=6). (**F**) Fatty acid abundance in conditioned medium from NFs or CAFs (HT15B and HT17B models) (*N*=4-5). (**G**) Fatty acid abundance in conditioned medium from NFs or CAFs (HT15B model) upon silica-based delipidation (*N*=2-5). (**H**) Viability of SC263 cells after treatment with 1 µg/mL cetuximab for 72h, in presence of CAF-CM (50% final volume; HT15B model) upon delipidation, with or without supplementation with chemically defined lipid concentrate (LC) (*N*=2-3; *n*=6). (**I-J**) Levels of EGF (**I**) and TGFα (**J**) in conditioned medium from NFs or CAFs (HT15B and HT17B models) upon silica-based delipidation (*N*=3). Data are plotted as the means ± SEM (**A-J**). *N* indicates the number of independent biological experiments and *n* indicates the number of technical replicates (when >1). Significance was determined by two-way ANOVA with Sidák’s multiple comparison test (**A-E,** and **G-J**), or one-way ANOVA with Dunnett’s multiple comparison test (**F**). *P*-values as indicated or ****P*<0.001; ns, not significant.

**Supplementary Figure 5. myCAF-derived FA support bioenergetic needs and EGFR signaling in HNSCC cells.** (**A-D**) Representative brightfield pictures (**A, C**) and quantification (**B, D**) of ORO-stained lipid droplets (LD) in HN5 (**A-B**) and SC263 cells (**C-D**) treated with 1 µg/mL cetuximab for 72 h in presence or not of (delipidated) conditioned medium from NFs and CAFs (HT15B model). Treatment with 50 µM BSA-conjugated oleic acid (OA) was used as positive control (*N*=3). (**E**) Basal oxygen consumption rate (OCR) in SC263 cells treated with (delipidated) conditioned medium from NFs and CAFs, in presence or not of 10 µM SSO (*N*=3; *n*=6). Data are plotted as the means ± SEM (**B** and **D-E**). *N* indicates the number of independent biological experiments and *n* indicates the number of technical replicates (when >1). Significance was determined by two-way ANOVA with Sidák’s multiple comparison test (**B** and **D**), or one-way ANOVA with Dunnett’s multiple comparison test (**E**). *P*-values as indicated or ****P*<0.001; ns, not significant.

**Supplementary Figure 6. Therapeutic targeting of tumor/stroma metabolic crosstalk impairs cetuximab resistance in HNSCC cells.** (**A**) Fatty acid abundance in conditioned medium from CAFs (HT15B model) treated or not with 10 µM TVB-3664 for 72 h (*N*=2-3). (**B-E**) Viability of HN5 and SC263 cells upon treatment with 1 µg/mL cetuximab in presence or not of conditioned medium from CAFs (HT15B model) pre-treated or not with 10 µM TVB-3664 (**B-C**) or 100 µM SSO (**D-E**) for 72 h (*N*=3; *n*=6). (**F**) Cell death levels in PDX-derived HNSCC organoids (UCLHN4 model) upon treatment with 2 µg/mL cetuximab for 2 weeks. (**G**) Viability of PDX-derived HNSCC organoids (UCLHN4 model) upon treatment with 2 µg/mL cetuximab in presence or not of delipidated CAF-CM (HT15B model) for 2 weeks (*N*=6). Data are plotted as the means ± SEM (**B-G**). *N* indicates the number of independent biological experiments and *n* indicates the number of technical replicates (when >1). Significance was determined by two-way ANOVA with Sidák’s multiple comparison test (**B-G**). *P*-values as indicated or ****P*<0.001; ns, not significant.

**Supplementary Figure 7. TGFβ2-dependent tumor-stroma metabolic crosstalk correlates with cetuximab tolerance in patient-derived HNSCC models.** (**A**) Proportion of read mapping to human or mouse genomes in RNA-seq data (GSE303798) from vehicle- and cetuximab-treated UCLHN4 PDX tissues (*N*=5-6). (**B-C**) mRNA expression levels of *TGFB1* (**B**) and *TGFB3* (**C**) in cetuximab-treated UCLHN4, #03 and #20 PDX models (*N*=3) (GSE303798 and GSE143279). (**D**) Relative mRNA expression (log_2_ fold-change) of *TGFB1*, *TGFB2* and *TGFB3* in cetuximab-resistant UCLHN4 and HNC004 PDX models (*N*=3) (GSE235417 and GSE307896). (**E-F**) Volcano plots depicting an enrichment for lipid metabolism-related genes in cetuximab-treated #03 (**E**) and #020 (**F**) PDX models (GSE142379) (*N*=3). (**G**) Body weight follow-up for UCLHN4 PDX-bearing nude mice treated with 30 mg/kg cetuximab, 50 mg/kg Trabedersen (for the first dose and then 16 mg/kg), alone or in combination. (**H**) GSEA plot of the OXPHOS-related gene signature in tiling array expression data (GSE109756) from cetuximab-treated HNSCC patients (post-cetuximab vs baseline) (*N* = 15). (**I**) Pre-cetuximab (baseline) TGF-β2 levels in plasma from HNSCC patients (alive or deceased after surgery) (*N*=15). Data are plotted as the means ± SEM (**A-C, G** and **I**). *N* indicates the number of independent biological experiments and *n* indicates the number of technical replicates (when >1). Significance was determined by two-way ANOVA with Sidák’s multiple comparison test (**A-C**), or two-tailed unpaired Student’s t-test (**I**). *P*-values as indicated or ****P*<0.001; ns, not significant.

**Supplementary Table 1. Sequences of human gene-specific primers for qRT-PCR used in this study.**

| **Gene name** | **Forward primer (5’ - 3’)** | **Reverse primer (5’ - 3’)** | **Amplicon length (bp)** |
| --- | --- | --- | --- |
| *CD36* | GGGAAAGTCACTGCGACATG | TGCAATACCTGGCTTTTCTCA | 79 |
| *CPT1A* | ATGTACGCCAAGATCGACCC | GACATGCAGTTGGCCGTTTC | 77 |
| *GTF2B* | ACTACAGAGCCGGTGATATGAT | GTTGCTTTGTCATTGCTGAAAGT | 106 |
| *TGFB2* | TGTCCCTGCTGCACTTTTGTA | GGTGCCATCAATACCTGCAAATC | 93 |
